# Examining empathy deficits across familial forms of frontotemporal dementia within the GENFI cohort

**DOI:** 10.1016/j.cortex.2022.01.012

**Published:** 2022-05

**Authors:** Phoebe H. Foster, Lucy L. Russell, Georgia Peakman, Rhian S. Convery, Arabella Bouzigues, Caroline V. Greaves, Martina Bocchetta, David M. Cash, John C. van Swieten, Lize C. Jiskoot, Fermin Moreno, Raquel Sanchez-Valle, Robert Laforce, Caroline Graff, Mario Masellis, Carmela Tartaglia, James B. Rowe, Barbara Borroni, Elizabeth Finger, Matthis Synofzik, Daniela Galimberti, Rik Vandenberghe, Alexandre de Mendonça, Chris R. Butler, Alex Gerhard, Simon Ducharme, Isabelle Le Ber, Fabrizio Tagliavini, Isabel Santana, Florence Pasquier, Johannes Levin, Adrian Danek, Markus Otto, Sandro Sorbi, Jonathan D. Rohrer, the Genetic FTD Initiative (GENFI), Sónia Afonso, Maria Rosario Almeida, Sarah Anderl-Straub, Christin Andersson, Anna Antonell, Silvana Archetti, Andrea Arighi, Mircea Balasa, Myriam Barandiaran, Nuria Bargalló, Robart Bartha, Benjamin Bender, Alberto Benussi, Maxime Bertoux, Anne Bertrand, Valentina Bessi, Sandra Black, Sergi Borrego-Ecija, Jose Bras, Alexis Brice, Rose Bruffaerts, Agnès Camuzat, Marta Cañada, Valentina Cantoni, Paola Caroppo, David Cash, Miguel Castelo-Branco, Olivier Colliot, Thomas Cope, Vincent Deramecourt, María de Arriba, Giuseppe Di Fede, Alina Díez, Diana Duro, Chiara Fenoglio, Camilla Ferrari, Catarina B. Ferreira, Nick Fox, Morris Freedman, Giorgio Fumagalli, Aurélie Funkiewiez, Alazne Gabilondo, Roberto Gasparotti, Serge Gauthier, Stefano Gazzina, Giorgio Giaccone, Ana Gorostidi, Caroline Greaves, Rita Guerreiro, Carolin Heller, Tobias Hoegen, Begoña Indakoetxea, Vesna Jelic, Hans-Otto Karnath, Ron Keren, Gregory Kuchcinski, Tobias Langheinrich, Thibaud Lebouvier, Maria João Leitão, Albert Lladó, Gemma Lombardi, Sandra Loosli, Carolina Maruta, Simon Mead, Lieke Meeter, Gabriel Miltenberger, Rick van Minkelen, Sara Mitchell, Katrina Moore, Benedetta Nacmias, Annabel Nelson, Linn Öijerstedt, Jaume Olives, Sebastien Ourselin, Alessandro Padovani, Jessica Panman, Janne M. Papma, Yolande Pijnenburg, Cristina Polito, Enrico Premi, Sara Prioni, Catharina Prix, Rosa Rademakers, Veronica Redaelli, Daisy Rinaldi, Tim Rittman, Ekaterina Rogaeva, Adeline Rollin, Pedro Rosa-Neto, Giacomina Rossi, Martin Rossor, Beatriz Santiago, Dario Saracino, Sabrina Sayah, Elio Scarpini, Sonja Schönecker, Harro Seelaar, Elisa Semler, Rachelle Shafei, Christen Shoesmith, Imogen Swift, Miguel Tábuas-Pereira, Mikel Tainta, Ricardo Taipa, David Tang-Wai, David L. Thomas, Paul Thompson, Hakan Thonberg, Carolyn Timberlake, Pietro Tiraboschi, Emily Todd, Philip Van Damme, Mathieu Vandenbulcke, Michele Veldsman, Ana Verdelho, Jorge Villanua, Jason Warren, Carlo Wilke, Ione Woollacott, Elisabeth Wlasich, Henrik Zetterberg, Miren Zulaica

**Affiliations:** as Instituto Ciencias Nucleares Aplicadas a Saude, Universidade de Coimbra, Coimbra, Portugal; at Faculty of Medicine, University of Coimbra, Coimbra, Portugal; au Department of Neurology, University of Ulm, Ulm, Germany; av Department of Clinical Neuroscience, Karolinska Institutet, Stockholm, Sweden; aw Alzheimer's Disease and Other Cognitive Disorders Unit, Neurology Service, Hospital Clínic, Barcelona, Spain; ax Biotechnology Laboratory, Department of Diagnostics, ASST Brescia Hospital, Brescia, Italy; ay Fondazione IRCCS Ca’ Granda Ospedale Maggiore Policlinico, Neurodegenerative Diseases Unit, Milan, Italy; az University of Milan, Centro Dino Ferrari, Milan, Italy; ba Alzheimer's Disease and Other Cognitive Disorders Unit, Neurology Service, Hospital Clínic, Barcelona, Spain; bb Cognitive Disorders Unit, Department of Neurology, Donostia University Hospital, San Sebastian, Gipuzkoa, Spain; bc Neuroscience Area, Biodonostia Health Research Institute, San Sebastian, Gipuzkoa, Spain; bd Imaging Diagnostic Center, Hospital Clínic, Barcelona, Spain; be Department of Medical Biophysics, The University of Western Ontario, London, Ontario, Canada; bf Centre for Functional and Metabolic Mapping, Robarts Research Institute, The University of Western Ontario, London, Ontario, Canada; bg Department of Diagnostic and Interventional Neuroradiology, University of Tübingen, Tübingen, Germany; bh Centre for Neurodegenerative Disorders, Department of Clinical and Experimental Sciences, University of Brescia, Italy; bi Inserm 1172, Lille, France; bk Sorbonne Université, Paris Brain Institute – Institut du Cerveau – ICM, Inserm U1127, CNRS UMR 7225, AP-HP - Hôpital Pitié-Salpêtrière, Paris, France; bl Inria, Aramis Project-team, F-75013, Paris, France; bm Centre pour l'Acquisition et le Traitement des Images, Institut du Cerveau et la Moelle, Paris, France; bn Department of Neuroscience, Psychology, Drug Research, and Child Health, University of Florence, Florence, Italy; bo Sunnybrook Health Sciences Centre, Sunnybrook Research Institute, University of Toronto, Toronto, Canada; bp Alzheimer's Disease and Other Cognitive Disorders Unit, Neurology Service, Hospital Clínic, Barcelona, Spain; bq Center for Neurodegenerative Science, Van Andel Institute, Grand Rapids, Michigan, MI, 49503, USA; br Sorbonne Université, Paris Brain Institute – Institut du Cerveau – ICM, Inserm U1127, CNRS UMR 7225, AP-HP - Hôpital Pitié-Salpêtrière, Paris, France; bs Reference Network for Rare Neurological Diseases (ERN-RND), Germany; bt Laboratory for Cognitive Neurology, Department of Neurosciences, KU Leuven, Leuven, Belgium; bu Sorbonne Université, Paris Brain Institute – Institut du Cerveau – ICM, Inserm U1127, CNRS UMR 7225, AP-HP - Hôpital Pitié-Salpêtrière, Paris, France; bv CITA Alzheimer, San Sebastian, Gipuzkoa, Spain; bw Centre for Neurodegenerative Disorders, Neurology Unit, Department of Clinical and Experimental Sciences, University of Brescia, Brescia, Italy; bx Fondazione IRCCS Istituto Neurologico Carlo Besta, Milano, Italy; by Dementia Research Centre, Department of Neurodegenerative Disease, UCL Institute of Neurology, Queen Square, London, UK; bz Faculty of Medicine, University of Coimbra, Coimbra, Portugal; ca Sorbonne Université, Paris Brain Institute – Institut du Cerveau – ICM, Inserm U1127, CNRS UMR 7225, AP-HP - Hôpital Pitié-Salpêtrière, Paris, France; cb Inria, Aramis Project-team, F-75013, Paris, France; cc Centre pour l'Acquisition et le Traitement des Images, Institut du Cerveau et la Moelle, Paris, France; cd Department of Clinical Neuroscience, University of Cambridge, Cambridge, UK; ce Univ Lille, France; cf Inserm 1172, Lille, France; cg CHU, CNR-MAJ, Labex Distalz, LiCEND, Lille, France; ch Neuroscience Area, Biodonostia Health Research Institute, San Sebastian, Gipuzkoa, Spain; ci Fondazione IRCCS Istituto Neurologico Carlo Besta, Milano, Italy; cj Neuroscience Area, Biodonostia Health Research Institute, San Sebastian, Gipuzkoa, Spain; ck Faculty of Medicine, University of Coimbra, Coimbra, Portugal; cl Fondazione IRCCS Ca’ Granda Ospedale Maggiore Policlinico, Neurodegenerative Diseases Unit, Milan, Italy; cm University of Milan, Centro Dino Ferrari, Milan, Italy; cn Department of Neuroscience, Psychology, Drug Research, and Child Health, University of Florence, Florence, Italy; co Laboratory of Neurosciences, Institute of Molecular Medicine, Faculty of Medicine, University of Lisbon, Lisbon, Portugal; cp Dementia Research Centre, Department of Neurodegenerative Disease, UCL Institute of Neurology, Queen Square, London, UK; cq Baycrest Health Sciences, Rotman Research Institute, University of Toronto, Toronto, Canada; cr Fondazione IRCCS Ca’ Granda Ospedale Maggiore Policlinico, Neurodegenerative Diseases Unit, Milan, Italy; cs University of Milan, Centro Dino Ferrari, Milan, Italy; ct Centre de référence des démences rares ou précoces, IM2A, Département de Neurologie, AP-HP - Hôpital Pitié-Salpêtrière, Paris, France; cu Sorbonne Université, Paris Brain Institute – Institut du Cerveau – ICM, Inserm U1127, CNRS UMR 7225, AP-HP - Hôpital Pitié-Salpêtrière, Paris, France; cv Neuroscience Area, Biodonostia Health Research Institute, San Sebastian, Gipuzkoa, Spain; cw Neuroradiology Unit, University of Brescia, Brescia, Italy; cx Alzheimer Disease Research Unit, McGill Centre for Studies in Aging, Department of Neurology & Neurosurgery, McGill University, Montreal, Québec, Canada; cy Neurology, ASST Brescia Hospital, Brescia, Italy; cz Fondazione IRCCS Istituto Neurologico Carlo Besta, Milano, Italy; da Neuroscience Area, Biodonostia Health Research Institute, San Sebastian, Gipuzkoa, Spain; db Dementia Research Centre, Department of Neurodegenerative Disease, UCL Institute of Neurology, Queen Square, London, UK; dc Center for Neurodegenerative Science, Van Andel Institute, Grand Rapids, Michigan, MI, 49503, USA; dd Dementia Research Centre, Department of Neurodegenerative Disease, UCL Institute of Neurology, Queen Square, London, UK; de Neurologische Klinik, Ludwig-Maximilians-Universität München, Munich, Germany; df Cognitive Disorders Unit, Department of Neurology, Donostia University Hospital, San Sebastian, Gipuzkoa, Spain; dg Neuroscience Area, Biodonostia Health Research Institute, San Sebastian, Gipuzkoa, Spain; dh Division of Clinical Geriatrics, Karolinska Institutet, Stockholm, Sweden; di Division of Neuropsychology, Hertie-Institute for Clinical Brain Research and Center of Neurology, University of Tübingen, Tübingen, Germany; dj The University Health Network, Toronto Rehabilitation Institute, Toronto, Canada; dk Univ Lille, France; dl Inserm 1172, Lille, France; dm CHU, CNR-MAJ, Labex Distalz, LiCEND Lille, France; dn Division of Neuroscience and Experimental Psychology, Wolfson Molecular Imaging Centre, University of Manchester, Manchester, UK; do Manchester Centre for Clinical Neurosciences, Department of Neurology, Salford Royal NHS Foundation Trust, Manchester, UK; dp Univ Lille, France; dq Inserm 1172, Lille, France; dr CHU, CNR-MAJ, Labex Distalz, LiCEND Lille, France; ds Centre of Neurosciences and Cell Biology, Universidade de Coimbra, Coimbra, Portugal; dt Alzheimer's Disease and Other Cognitive Disorders Unit, Neurology Service, Hospital Clínic, Barcelona, Spain; du Department of Neuroscience, Psychology, Drug Research and Child Health, University of Florence, Florence, Italy; dv Neurologische Klinik, Ludwig-Maximilians-Universität München, Munich, Germany; dw Laboratory of Language Research, Centro de Estudos Egas Moniz, Faculty of Medicine, University of Lisbon, Lisbon, Portugal; dx MRC Prion Unit, Department of Neurodegenerative Disease, UCL Institute of Neurology, Queen Square, London, UK; dy Department of Neurology, Erasmus Medical Center, Rotterdam, Netherlands; dz Faculty of Medicine, University of Lisbon, Lisbon, Portugal; ea Department of Clinical Genetics, Erasmus Medical Center, Rotterdam, Netherlands; eb Sunnybrook Health Sciences Centre, Sunnybrook Research Institute, University of Toronto, Toronto, Canada; ec Dementia Research Centre, Department of Neurodegenerative Disease, UCL Institute of Neurology, Queen Square, London, UK; ed Department of Neuroscience, Psychology, Drug Research and Child Health, University of Florence, Florence, Italy; ee Dementia Research Centre, Department of Neurodegenerative Disease, UCL Institute of Neurology, Queen Square, London, UK; ef Center for Alzheimer Research, Division of Neurogeriatrics, Department of Neurobiology, Care Sciences and Society, Bioclinicum, Karolinska Institutet, Solna, Sweden; eg Unit for Hereditary Dementias, Theme Aging, Karolinska University Hospital, Solna, Sweden; eh Alzheimer's Disease and Other Cognitive Disorders Unit, Neurology Service, Hospital Clínic, Barcelona, Spain; ei School of Biomedical Engineering & Imaging Sciences, King's College London, London, UK; ej Centre for Neurodegenerative Disorders, Department of Clinical and Experimental Sciences, University of Brescia, Italy; ek Department of Neurology, Erasmus Medical Center, Rotterdam, Netherlands; el Department of Neurology, Erasmus Medical Center, Rotterdam, Netherlands; em Amsterdam University Medical Centre, Amsterdam VUmc, Amsterdam, Netherlands; en Department of Biomedical, Experimental and Clinical Sciences “Mario Serio”, Nuclear Medicine Unit, University of Florence, Florence, Italy; eo Stroke Unit, ASST Brescia Hospital, Brescia, Italy; ep Fondazione IRCCS Istituto Neurologico Carlo Besta, Milano, Italy; eq Neurologische Klinik, Ludwig-Maximilians-Universität München, Munich, Germany; er Department of Neurosciences, Mayo Clinic, Jacksonville, FL, USA; es Fondazione IRCCS Istituto Neurologico Carlo Besta, Milano, Italy; et Centre de référence des démences rares ou précoces, IM2A, Département de Neurologie, AP-HP - Hôpital Pitié-Salpêtrière, Paris, France; eu Sorbonne Université, Paris Brain Institute – Institut du Cerveau – ICM, Inserm U1127, CNRS UMR 7225, AP-HP - Hôpital Pitié-Salpêtrière, Paris, France; ev Département de Neurologie, AP-HP - Hôpital Pitié-Salpêtrière, Paris, France; ew Reference Network for Rare Neurological Diseases (ERN-RND), Germany; ex Department of Clinical Neurosciences, University of Cambridge, Cambridge, UK; ey Tanz Centre for Research in Neurodegenerative Diseases, University of Toronto, Toronto, Canada; ez CHU, CNR-MAJ, Labex Distalz, LiCEND, Lille, France; fa Translational Neuroimaging Laboratory, McGill Centre for Studies in Aging, McGill University, Montreal, Québec, Canada; fb Fondazione IRCCS Istituto Neurologico Carlo Besta, Milano, Italy; fc Dementia Research Centre, Department of Neurodegenerative Disease, UCL Institute of Neurology, Queen Square, London, UK; fd Neurology Department, Centro Hospitalar e Universitario de Coimbra, Coimbra, Portugal; fe Sorbonne Université, Paris Brain Institute – Institut du Cerveau – ICM, Inserm U1127, CNRS UMR 7225, AP-HP - Hôpital Pitié-Salpêtrière, Paris, France; ff Inria, Aramis Project-team, F-75013, Paris, France; fg Centre de référence des démences rares ou précoces, IM2A, Département de Neurologie, AP-HP - Hôpital Pitié-Salpêtrière, Paris, France; fh Sorbonne Université, Paris Brain Institute – Institut du Cerveau – ICM, Inserm U1127, CNRS UMR 7225, AP-HP - Hôpital Pitié-Salpêtrière, Paris, France; fi Fondazione IRCCS Ca’ Granda Ospedale Maggiore Policlinico, Neurodegenerative Diseases Unit, Milan, Italy; fj University of Milan, Centro Dino Ferrari, Milan, Italy; fk Neurologische Klinik, Ludwig-Maximilians-Universität München, Munich, Germany; fl Department of Neurology, Erasmus Medical Centre, Rotterdam, Netherlands; fm Department of Neurology, University of Ulm, Ulm, Germany; fn Dementia Research Centre, Department of Neurodegenerative Disease, UCL Institute of Neurology, Queen Square, London, UK; fo Department of Clinical Neurological Sciences, University of Western Ontario, London, Ontario, Canada; fp Department of Neurodegenerative Disease, Dementia Research Centre, UCL Institute of Neurology, Queen Square, London, UK; fq Neurology Department, Centro Hospitalar e Universitario de Coimbra, Coimbra, Portugal; fr Neuroscience Area, Biodonostia Health Research Institute, San Sebastian, Gipuzkoa, Spain; fs Neuropathology Unit and Department of Neurology, Centro Hospitalar do Porto - Hospital de Santo António, Oporto, Portugal; ft The University Health Network, Krembil Research Institute, Toronto, Canada; fu Neuroimaging Analysis Centre, Department of Brain Repair and Rehabilitation, UCL Institute of Neurology, Queen Square, London, UK; fv Division of Neuroscience and Experimental Psychology, Wolfson Molecular Imaging Centre, University of Manchester, Manchester, UK; fw Center for Alzheimer Research, Division of Neurogeriatrics, Karolinska Institutet, Stockholm, Sweden; fx Department of Clinical Neurosciences, University of Cambridge, Cambridge, UK; fy Fondazione IRCCS Istituto Neurologico Carlo Besta, Milano, Italy; fz Dementia Research Centre, Department of Neurodegenerative Disease, UCL Institute of Neurology, UK; ga Neurology Service, University Hospitals Leuven, Belgium; gb Laboratory for Neurobiology, VIB-KU Leuven Centre for Brain Research, Leuven, Belgium; gc Geriatric Psychiatry Service, University Hospitals Leuven, Belgium; ge Nuffield Department of Clinical Neurosciences, Medical Sciences Division, University of Oxford, Oxford, UK; gf Department of Neurosciences and Mental Health, Centro Hospitalar Lisboa Norte - Hospital de Santa Maria & Faculty of Medicine, University of Lisbon, Lisbon, Portugal; gg OSATEK, University of Donostia, San Sebastian, Gipuzkoa, Spain; gh Dementia Research Centre, Department of Neurodegenerative Disease, UCL Institute of Neurology, Queen Square, London, UK; gi Department of Neurodegenerative Diseases, Hertie-Institute for Clinical Brain Research and Center of Neurology, University of Tübingen, Tübingen, Germany; gj Center for Neurodegenerative Diseases (DZNE), Tübingen, Germany; gk Dementia Research Centre, Department of Neurodegenerative Disease, UCL Institute of Neurology, Queen Square, London, UK; gl Neurologische Klinik, Ludwig-Maximilians-Universität München, Munich, Germany; gm Dementia Research Institute, Department of Neurodegenerative Disease, UCL Institute of Neurology, Queen Square, London, UK; gn Neuroscience Area, Biodonostia Health Research Institute, San Sebastian, Gipuzkoa, Spain; bj CHU, CNR-MAJ, Labex Distalz, LiCEND, Lille, France; gd Neuropsychiatry, Department of Neurosciences, KU Leuven, Leuven, Belgium; a Dementia Research Centre, Department of Neurodegenerative Disease, UCL Queen Square Institute of Neurology, London, UK; b Centre for Medical Image Computing, University College London, London, UK; c Department of Neurology, Erasmus Medical Centre, Rotterdam, Netherlands; d Cognitive Disorders Unit, Department of Neurology, Donostia Universitary Hospital, San Sebastian, Spain; e Neuroscience Area, Biodonostia Health Research Institute, San Sebastian, Gipuzkoa, Spain; f Alzheimer's Disease and Other Cognitive Disorders Unit, Neurology Service, Hospital Clínic, Institut d’Investigacións Biomèdiques August Pi I Sunyer, University of Barcelona, Barcelona, Spain; g Clinique Interdisciplinaire de Mémoire, Département des Sciences Neurologiques, CHU de Québec, and Faculté de Médecine, Université Laval, QC, Canada; h Center for Alzheimer Research, Division of Neurogeriatrics, Department of Neurobiology, Care Sciences and Society, Bioclinicum, Karolinska Institutet, Solna, Sweden; i Unit for Hereditary Dementias, Theme Aging, Karolinska University Hospital, Solna, Sweden; j Sunnybrook Health Sciences Centre, Sunnybrook Research Institute, University of Toronto, Toronto, Canada; k Tanz Centre for Research in Neurodegenerative Diseases, University of Toronto, Toronto, ON, Canada; l University of Cambridge Department of Clinical Neurosciences, and University of Cambridge Hospitals NHS Trust, University of Cambridge, UK; m Neurology Unit, Department of Clinical and Experimental Sciences, University of Brescia, Brescia, Italy; n Department of Clinical Neurological Sciences, University of Western Ontario, London, ON, Canada; o Department of Neurodegenerative Diseases, Hertie-Institute for Clinical Brain Research and Center of Neurology, University of Tübingen, Tübingen, Germany; p Center for Neurodegenerative Diseases (DZNE), Tübingen, Germany; q Fondazione Ca’ Granda, IRCCS Ospedale Policlinico, Milan, Italy; r University of Milan, Centro Dino Ferrari, Milan, Italy; s Laboratory for Cognitive Neurology, Department of Neurosciences, KU Leuven, Leuven, Belgium; t Neurology Service, University Hospitals Leuven, Leuven, Belgium; u Leuven Brain Institute, KU Leuven, Leuven, Belgium; v Faculty of Medicine, University of Lisbon, Lisbon, Portugal; w Nuffield Department of Clinical Neurosciences, Medical Sciences Division, University of Oxford, Oxford, UK; x Department of Brain Sciences, Imperial College London, UK; y Division of Neuroscience and Experimental Psychology, Wolfson Molecular Imaging Centre, University of Manchester, Manchester, UK; z Departments of Geriatric Medicine and Nuclear Medicine, University of Duisburg-Essen, Germany; aa Department of Psychiatry, McGill University Health Centre, McGill University, Montreal, Québec, Canada; ab McConnell Brain Imaging Centre, Montreal Neurological Institute, McGill University, Montreal, Québec, Canada; ac Sorbonne Université, Paris Brain Institute – Institut du Cerveau – ICM, Inserm U1127, CNRS UMR 7225, AP-HP - Hôpital Pitié-Salpêtrière, Paris, France; ad Centre de référence des démences rares ou précoces, IM2A, Département de Neurologie, AP-HP - Hôpital Pitié-Salpêtrière, Paris, France; ae Département de Neurologie, AP-HP - Hôpital Pitié-Salpêtrière, Paris, France; af Reference Network for Rare Neurological Diseases (ERN-RND); ag Fondazione IRCCS Istituto Neurologico Carlo Besta, Milano, Italy; ah University Hospital of Coimbra (HUC), Neurology Service, Faculty of Medicine, University of Coimbra, Coimbra, Portugal; ai Center for Neuroscience and Cell Biology, Faculty of Medicine, University of Coimbra, Coimbra, Portugal; aj Univ Lille, France; ak Inserm 1172, Lille, France; al CHU, CNR-MAJ, Labex Distalz, LiCEND Lille, France; am Department of Neurology, Ludwig-Maximilians Universität München, Munich, Germany; an German Center for Neurodegenerative Diseases (DZNE), Munich, Germany; ao Munich Cluster of Systems Neurology (SyNergy), Munich, Germany; ap Department of Neurology, University of Ulm, Germany; aq Department of Neurofarba, University of Florence, Italy; ar IRCCS Fondazione Don Carlo Gnocchi, Florence, Italy

**Keywords:** Empathy, Frontotemporal dementia, Perspective taking, Empathic concern, Interpersonal Reactivity Index

## Abstract

**Background:**

Reduced empathy is a common symptom in frontotemporal dementia (FTD). Although empathy deficits have been extensively researched in sporadic cases, few studies have explored the differences in familial forms of FTD.

**Methods:**

Empathy was examined using a modified version of the Interpersonal Reactivity Index (mIRI) in 676 participants from the Genetic FTD Initiative: 216 mutation-negative controls, 192 *C9orf72* expansion carriers, 193 *GRN* mutation carriers and 75 *MAPT* mutation carriers. Using global scores from the CDR® plus NACC FTLD, mutation carriers were divided into three groups, asymptomatic (0), very mildly symptomatic/prodromal (.5), or fully symptomatic (1 or more). The mIRI Total score, as well as the subscores of Empathic Concern (EC) and Perspective Taking (PT) were assessed. Linear regression models with bootstrapping were used to assess empathy ratings across genetic groups, as well as across phenotypes in the symptomatic carriers. Neural correlates of empathy deficits were examined using a voxel-based morphometry (VBM) analysis.

**Results:**

All fully symptomatic groups scored lower on the mIRI Total, EC, and PT when compared to controls and their asymptomatic or prodromal counterparts (all *p* < .001). Prodromal *C9orf72* expansion carriers also scored significantly lower than controls on the mIRI Total score (*p* = .046). In the phenotype analysis, all groups (behavioural variant FTD, primary progressive aphasia and FTD with amyotrophic lateral sclerosis) scored significantly lower than controls (all *p* < .007). VBM revealed an overlapping neural correlate of the mIRI Total score across genetic groups in the orbitofrontal lobe but with additional involvement in the temporal lobe, insula and basal ganglia in both the *GRN* and *MAPT* groups, and uniquely more posterior regions such as the parietal lobe and thalamus in the *GRN* group, and medial temporal structures in the *MAPT* group.

**Conclusions:**

Significant empathy deficits present in genetic FTD, particularly in symptomatic individuals and those with a bvFTD phenotype, while prodromal deficits are only seen using the mIRI in *C9orf72* expansion carriers.

## Abbreviations

FTD: frontotemporal dementia
mIRI: modified Interpersonal Reactivity Index
C9orf72: chromosome 9 open reading frame 72
GRN: progranulin
MAPT: microtubule associated protein tau
CDR plus NACC FTLD: CDR® Dementia Staging Instrument with National Alzheimer Coordinating Centre Frontotemporal Lobar Degeneration component
EC: empathic concern
PT: perspective taking
VBM: voxel-based morphometry
bvFTD: behavioural variant frontotemporal dementia
PPA: primary progressive aphasia
FTD-ALS: frontotemporal dementia with amyotrophic lateral sclerosis
GENFI: the Genetic Frontotemporal dementia Initiative
MMSE: Mini-Mental State Examination
SB: sum of boxes
MRI: magnetic resonance imaging
SPM: statistical parametric mapping
MNI: Montreal Neurological Institute
TIV: total intracranial volume
FWE: family wise error
IRI: Interpersonal Reactivity Index
SD: standard deviation

## 1 Introduction

Frontotemporal dementia (FTD) is a heterogenous group of neurodegenerative disorders, characterised by predominant atrophy in the frontal and temporal lobes (Rohrer et al., 2015; Seelaar, Rohrer, Pijnenburg, Fox & van Swieten, 2011; Warren, Rohrer & Rossor, 2013). The disease spectrum encompasses a variety of clinical syndromes: behavioural variant FTD (bvFTD), identifiable by altered personality and behavioural change, as well as a number of language variants, collectively referred to as primary progressive aphasia (PPA), distinguished by progressive deficits in word retrieval, comprehension or speech production (Calabria, Cotelli, Adenzato, Zanetti & Miniussi, 2009; Chow, Miller, Hayashi & Geschwind, 1999; Deleon & Miller, 2018; Englund et al., 1994; Farb et al., 2013; Marshall et al., 2018; Rohrer et al., 2009; Seelaar et al., 2011; Strong et al., 2009). Overlapping motor syndromes such as FTD with amyotrophic lateral sclerosis (FTD-ALS) also occur within the spectrum (Lashley, Rohrer, Mead & Revesz, 2015; Savage et al., 2014; Strong et al., 2009; Woollacott & Rohrer, 2016).

FTD is a highly heritable disease with around 20–30% of cases inherited via autosomal dominant transmission (Jiskoot et al., 2018; Lashley et al., 2015; Rohrer, Warren, Fox & Rossor, 2013; Rohrer et al., 2015). Mutations in one of three genes account for the majority of familial FTD: microtubule associated protein tau (*MAPT*), progranulin (*GRN*) and chromosome 9 open reading frame 72 (*C9orf72*) (Greaves & Rohrer, 2019; Tsvetanov et al., 2020; Warren, Rohrer & Rossor, 2013; Whitwell et al., 2012). Each account for around 5–10% of FTD cases, though geographic variability has been described (Deleon & Miller, 2018; Greaves & Rohrer, 2019; Rohrer & et al., 2009, 2013, 2015).

Despite various underlying pathologies, contributory genes, and clinical presentations, abnormal behaviours and social-emotional dysfunction are central to FTD syndromes, with a diminished capacity for empathy presenting as a core clinical symptom (Baez et al., 2014; Fittipaldi et al., 2019; Gorno-Tempini et al., 2011; Hazelton, Irish, Hodges, Piguet & Kumfor, 2017; Hsieh, Irish, Daveson, Hodges & Piguet, 2013; Lashley, Rohrer, Mead & Revesz, 2015; Kumfor et al., 2016; Savage et al., 2014). Empathy is widely recognised as the ability to ‘put oneself in another's shoes’, interpret others' emotions and appropriately respond to their experience (Calabria et al., 2009; Chrysikou & Thompson, 2016; Hazelton et al., 2017). Accordingly, it forms a fundamental aspect of social relatedness, facilitating the formation of strong foundations with those in one's social environment (Bernhardt & Singer, 2012; Chrysikou & Thompson, 2016; Perry et al., 2001; Rankin et al., 2006). Manifesting as interpersonal coldness and a reduced social interest, the empathy deficits seen in people with FTD are arguably one of the most distressing behaviours experienced by relatives and caregivers, demonstrating the need for clinical research (Baez & et al., 2014; Rankin et al., 2006; Snowden, 2018).

Empathy is conceptualised as a multidimensional construct, regulated by unconscious affective and conscious cognitive processes (Bernhardt & Singer, 2012; Cuff, Brown, Taylor & Howat, 2016; Davis, 1980). The Interpersonal Reactivity Index (Davis, 1980) is a psychometric measure, developed to reliably quantify this multifaceted construct. Given that limited self-awareness and a loss of insight into one's social functioning is commonplace in neurodegenerative diseases (Eslinger et al., 2005; Eslinger, Moore, Anderson & Grossman, 2011; Rankin et al., 2006; Viskontas, Possin & Miller, 2007; Woollacott & Rohrer, 2016)**,** a modified version of the index (hereafter referred to as the mIRI) was developed and used in the present study. The modified index ensures that a more accurate reflection of real-life empathic behaviours can be collected, by enabling third-party informants to respond on behalf of participants, thus overcoming issues of patient anosognosia (Fernandez-Duque, Hodges, Baird & Black, 2010; Hsieh et al., 2013; Lough et al., 2006).

Though empathy deficits and their neural correlates have been extensively researched in sporadic FTD (Eslinger et al., 2011; Rankin, Kramer & Miller, 2005; Rankin et al., 2006)**,** few studies have examined the possible differences between familial FTD groups. In the present study therefore, the mIRI was employed alongside a whole-brain voxel-based morphometry analysis with the aim of: i) exploring empathy in familial FTD groups; ii) evaluating the use of the mIRI as a measure of social cognitive change at different stages of symptom progression; and iii) elucidating the neural networks of total, affective, and cognitive empathy in genetic FTD. Due to genetic mutation-specific neurodegeneration (Cash et al., 2018; Rohrer et al., 2015), it was hypothesised that each familial group would present with divergent but overlapping neural networks associated with empathy. Furthermore, it was predicted that empathy would start to diminish as the disease progressed.

## 2 Methods

### 2.1 Participants

A total of 844 participants were recruited from the fifth data freeze of the Genetic FTD Initiative (GENFI) study, incorporating participant data from 27 sites. Of those enlisted, 676 had existing cross-sectional mIRI scores as completed by their informant, including 216 mutation-negative healthy controls, 192 *C9orf72* expansion carriers, 193 *GRN* mutation carriers and 75 *MAPT* mutation carriers (Table 1). Ethical approval was gained locally at each GENFI site and all participants provided informed written consent.

**Table 1:** Participant demographics including mean and standard deviation (in parentheses) scores for age at visit, years spent in education, as well as Mini-Mental State Examination (MMSE), CDR plus NACC FTLD sum of boxes (SB), and the modified Interpersonal Reactivity Index (mIRI) scores (Total, EC = Empathic Concern, PT = Perspective Taking). N equals the number of participants. Significant differences for sex (chi-squared test), age (linear regression) and education (linear regression) are shown in the table where a ∗ indicates a *p*-value of less than .05 comparing the disease group and controls.

	CDR plus NACC FTLD - global	N	% Male	Age	Education	MMSE	CDR plus NACC FTLD-SB	mIRI Total	mIRI EC	mIRI PT
Controls	0	216	40	45.7 (13.0)	14.3 (3.3)	29.3 (1.1)	.0 (.0)	53.0 (9.5)	27.6 (5.2)	25.4 (5.4)
*C9orf72*	0	94	41	43.9∗ (11.6)	14.3 (3.0)	29.1 (1.2)	.0 (.0)	50.7 (10.0)	26.3 (5.9)	24.4 (5.5)
	.5	33	45	49.9 (11.3)	13.9 (2.7)	28.4 (2.2)	1.1 (.7)	48.5 (12.4)	25.6 (6.2)	22.8 (7.7)
	1+	65	66∗	62.9∗ (9.5)	13.0∗ (3.6)	23.2 (6.8)	11.1 (5.6)	36.7 (10.5)	21.3 (6.4)	15.4 (5.3)
*GRN*	0	121	33	45.9 (12.1)	14.7 (3.4)	29.5 (.8)	.0 (.0)	53.0 (8.1)	27.3 (5.1)	25.7 (4.8)
	.5	25	44	51.4∗ (13.6)	14.0 (4.2)	28.6 (2.3)	1.0 (.8)	51.1 (12.8)	27.0 (6.6)	24.1 (7.4)
	1+	47	47	63.0∗ (7.4)	11.7∗ (3.4)	20.1 (7.7)	9.8 (6.2)	38.3 (11.4)	21.3 (6.1)	17.0 (6.1)
*MAPT*	0	41	39	38.6 (11.2)	14.5 (3.3)	29.5 (.8)	.0 (.0)	50.9 (10.5)	26.6 (5.5)	24.3 (6.1)
	.5	13	31	46.4 (12.8)	13.6 (2.5)	28.1 (2.3)	1.1 (.8)	53.7 (10.5)	28.3 (6.1)	25.4 (6.0)
	1+	21	57	58.9∗ (9.4)	13.6 (4.0)	21.9 (8.1)	10.3 (6.0)	34.6 (13.7)	20.0 (8.3)	14.6 (6.8)

Following the standardised GENFI protocol, a clinical examination was conducted on all participants, comprising of a physical examination, assessment of family and medical history, the Mini-Mental State Examination (MMSE), and the CDR® Dementia Staging Instrument with National Alzheimer Coordinating Centre Frontotemporal Lobar Degeneration component (CDR® plus NACC FTLD). The CDR® plus NACC FTLD measures functional, cognitive, language, and behavioural domains to generate a sum of boxes score (SB) and a global score, reflective of disease severity. Using these CDR® plus NACC FTLD global scores, mutation carriers were classified as either asymptomatic, very mildly symptomatic/prodromal, or fully symptomatic, corresponding to a value of 0, .5, or ≥1 (1+ i.e., those scoring 1, 2 or 3), respectively (Table 1). For inclusion as a healthy control in the analysis, a CDR® plus NACC FTLD-SB and global score of zero was required. In the 133 symptomatic participants (CDR 1+), clinical diagnoses (as per standard diagnostic criteria (Gorno-Tempini et al., 2011; Rascovsky et al., 2011; Strong et al., 2017)) were as follows: *C9orf72*, bvFTD = 48, PPA = 3, FTD-ALS = 7, Other = 7; *GRN*, bvFTD = 25, PPA = 17, Other = 5; *MAPT*, bvFTD = 17, PPA = 1, Other = 3. Demographics of all participants are displayed in Table 1.

### 2.2 The modified Interpersonal Reactivity Index

The modified version of the IRI that we used here is a 14-item questionnaire consisting of two seven-item subscales that measure separate empathy components (Rankin et al., 2006). The Perspective Taking (PT) scale measures the tendency to spontaneously consider or adopt another person's viewpoint, referred to as a *cognitive* facet of empathy**.** The Empathic Concern (EC) scale assesses feelings of sympathy and concern in response to another person's negative emotional state (Fernandez-Duque et al., 2010; Rankin et al., 2005; Shany-Ur et al., 2012), regarded as an *affective* facet of empathy. Each item in the scale is scored from 1 to 5, with five reverse-oriented questions included to deter response bias. Informants provided ratings reflecting participants current behaviour, with a total score (namely the mIRI Total, out of 70) and two component subscores (the mIRI EC and mIRI PT, out of 35 each) measured. A lower score on the mIRI corresponds to less empathy.

### 2.3 Statistical analysis

All statistical analyses were conducted using Stata/IC (version 16.1). In the healthy control group, Spearman rank correlations were conducted to determine the influence of age and years of education on the mIRI Total scores, whilst a Mann Whitney-U test was employed to explore the relationship between controls’ mIRI Total scores and sex.

The mIRI Total, PT, and EC scores were assessed across the genetic groups using linear regression models, with 95% bootstrapped confidence intervals (2000 repetitions) as the data was not normally distributed, adjusting for sex.

In addition, bootstrapped linear regression analyses were performed to compare the mIRI Total, EC, and PT scores between the symptomatic phenotype groups (bvFTD, PPA and FTD-ALS) and controls, adjusting for sex.

Correlation analyses were conducted across the familial groups to determine the relationship between mIRI Total score and disease severity (defined using CDR® plus NACC FTLD-SB scores).

### 2.4 Imaging analysis

Participants underwent volumetric T1-weighted magnetic resonance imaging (MRI) in a 3T scanner, as per the standardized GENFI protocol. Images containing motion or scanning artefacts were removed during a quality-control check. Further exclusions from the analysis were made if individuals displayed moderate to severe vascular disease or had an incidental space occupying lesion. Subsequently, 370 scans were included in the analysis (GE (2), Philips Achieva (120), Siemens Prisma (77), Siemens Skyra (66), or Siemens Trio (105)): 149 *C9orf72* expansion carriers, 161 *GRN* mutation carriers, and 60 *MAPT* mutation carriers. Control participants were not included in this stage of analysis.

Voxel-based morphometry (VBM) analysis was then performed using Statistical Parametric Mapping (SPM) 12, version 7219 (www.fil.ion.ucl.ac.uk/spm), running under Matlab R2014b (Mathworks, USA). The T1-weighted images were normalised and segmented into tissue type (grey matter, white matter, and cerebrospinal fluid) probability maps utilising a standard procedure and a fast-diffeomorphic image registration algorithm (DARTEL) (Ashburner, 2007). Grey matter segmentations were then normalised to Montreal Neurological Institute (MNI) space, smoothed and modulated using a Gaussian kernel with 6 mm full-width at half maximum, followed by application of a mask (Ridgway et al., 2009)**.** Total intracranial volume (TIV) was calculated using SPM (Malone et al., 2015).

To elucidate the neural correlates of empathy across familial FTD groups, flexible factorial regression models were performed, examining the relationship between grey matter volume and each component score of empathy (Total, PT, and EC scores). Genetic group and scanner type were used as factors in the analysis with age at scan, sex, disease severity (as measured using the CDR® plus NACC FTLD-SB score) and TIV included as covariates in the statistical model. The Family-Wise Error (FWE) rate for multiple comparisons correction was set at .05. If there were no findings at that strict level of correction, results were reviewed at an uncorrected *p* value of .001.

## 3 Results

### 3.1 Healthy controls

The mIRI Total scores in healthy controls ranged from 29 to 70 out of a possible maximum of 70. Cumulative frequency is displayed in Table S1, with a score of below 38 marking the 5th percentile cut-off.

No significant correlations were found between the mIRI Total scores and age (rho = .10, *p* = .240) or education (rho = .10, *p* = .245) in the control group. However, the effect of sex was found to be significant (*p* = .032) with female controls acquiring higher scores than their male counterparts (n = 129, mean 54.2 (standard deviation 9.3); n = 87, 51.3 (9.7), respectively). Table S2 shows the scores by decade in the total group and separately in each sex.

### 3.2 Mutation carriers

**Fig. 1:**
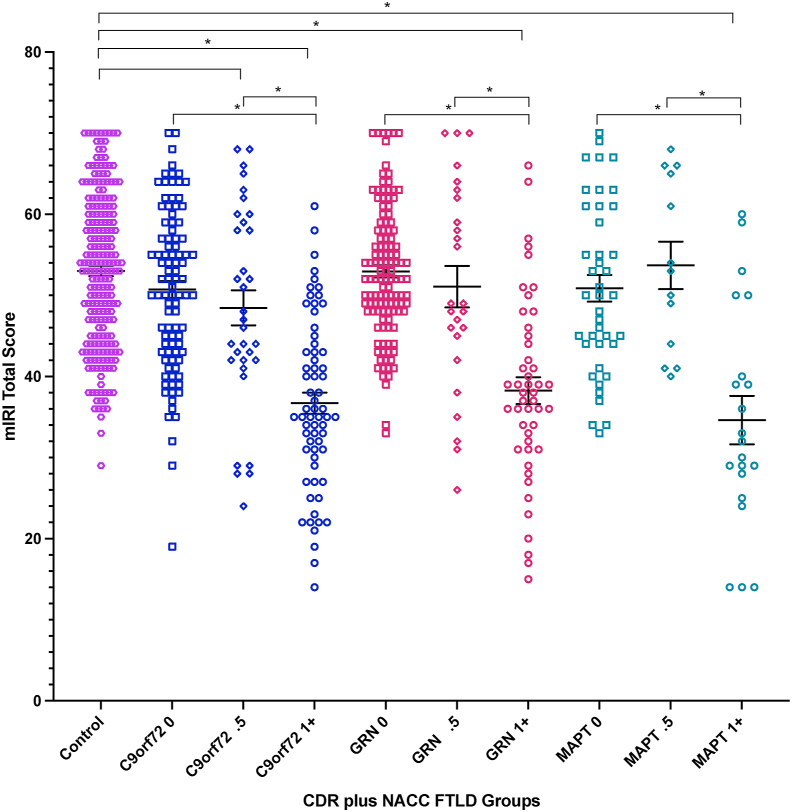
Modified Interpersonal Reactivity Index (mIRI) Total scores in each genetic group stratified by CDR plus NACC FTLD (0 = asymptomatic, .5 = mildly symptomatic/prodromal, 1+ = fully symptomatic). Means and standard errors are shown. Significant differences from controls and within groups are starred (p < .05). Lower score corresponds to less empathy.

**Fig. 2:**
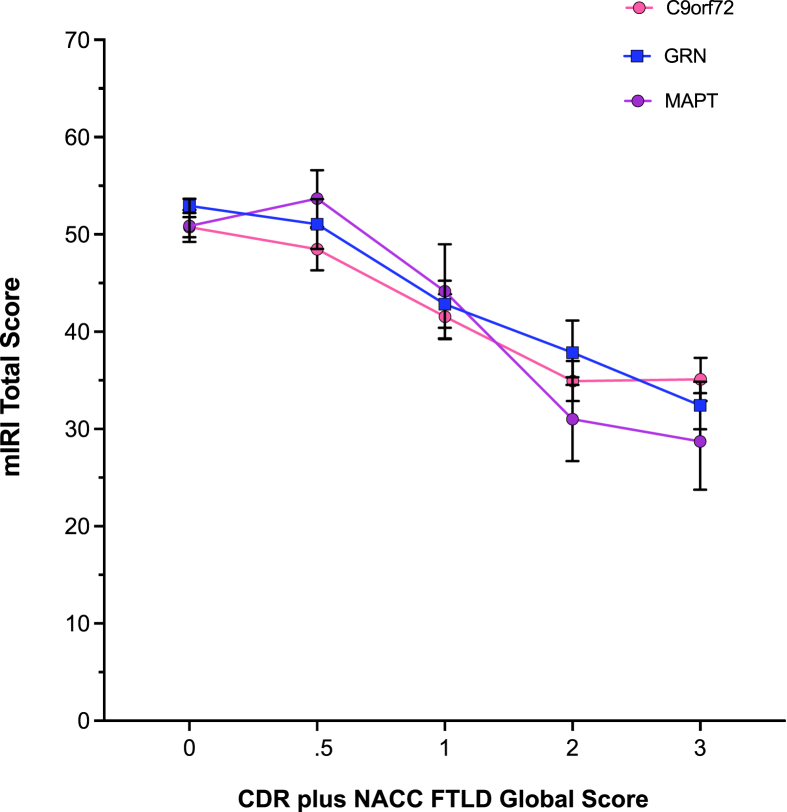
Modified Interpersonal Reactivity Index (mIRI) mean Total score with standard errors shown in each genetic group by CDR plus NACC FTLD global score.

All three fully symptomatic groups (CDR 1+) scored significantly worse than controls on all measures of empathy (mIRI Total, PT and EC scores, all *p* < .001) (Fig. 1, Fig. 2 and S1, Table 1). In the very mildly symptomatic/prodromal mutation carriers (CDR .5), only the *C9orf72* group showed a difference compared to controls with empathy ratings on the mIRI Total score significantly lower (*p* = .046). By contrast, when comparing the other prodromal (CDR .5) or asymptomatic (CDR 0) genetic groups with controls, no significant differences were observed (all *p* > .05). See Table S3 (Total score), Table S4 (EC subscore), and Table S5 (PT subscore) for all linear regression results.

Comparisons within genetic groups revealed that symptomatic carriers scored significantly lower than both very mildly symptomatic and asymptomatic participants on each empathy score (all *p* < .001) (Fig. 1 and S1). No differences between the very mildly symptomatic and asymptomatic participants were seen within any of the groups (Fig. 1 and S1).

No significant differences were observed between the symptomatic mutation carriers between the different genetic groups (or across either the very mildly symptomatic or asymptomatic mutation carriers).

### 3.3 Phenotype analysis

All three phenotypes (bvFTD, PPA and FTD-ALS) scored significantly lower than controls on all empathy scores (all *p* < .010) (Table 2 and S6). BvFTD participants scored significantly lower on the mIRI Total (*p* < .001), mIRI EC (*p* = .002), and mIRI PT (*p* < .001) scores than the PPA group. Furthermore, FTD-ALS participants scored significantly lower than the PPA group on the mIRI Total and mIRI PT scores (*p* = .012; *p* = .005 respectively). No other significant differences between the phenotype groups were found.

**Table 2:** Mean (M) and standard deviation (SD) mIRI Total, Empathic Concern (EC), and Perspective Taking (PT) scores for phenotypic groups (behavioural variant frontotemporal dementia, bvFTD, primary progressive aphasia, PPA, frontotemporal dementia with amyotrophic lateral sclerosis, FTD-ALS) and controls.

Diagnosis	mIRI Total		mIRI EC		mIRI PT	
	M	SD	M	SD	M	SD
Controls	53.0	9.5	27.6	5.2	25.4	5.4
bvFTD	33.9	11.0	19.5	6.6	14.4	5.4
PPA	43.8	9.6	24.2	5.5	19.6	5.8
FTD-ALS	35.3	5.5	21.9	3.2	13.4	4.5

### 3.4 Correlations with disease severity

Analyses across the genetic groups revealed negative correlations between disease severity and the mIRI Total score (*C9orf72*: rho = −.51, *p* < .001; *GRN*: rho = −.50, *p* < .001; *MAPT*: rho = −.46, *p* < .001), the mIRI EC subscore (*C9orf72*: rho = −.33, *p* < .001; *GRN*: rho = −.33, *p* < .001; *MAPT*: rho = −.29, *p* = .002), and the mIRI PT subscore (*C9orf72*: rho = −.52, *p* < .001; *GRN*: rho = −.43, *p* < .001; *MAPT*: rho = −.44, *p* < .001).

### 3.5 Imaging analysis

Significant relationships were observed between grey matter volume and the mIRI Total score in each of the genetic groups.

**Fig. 3:**
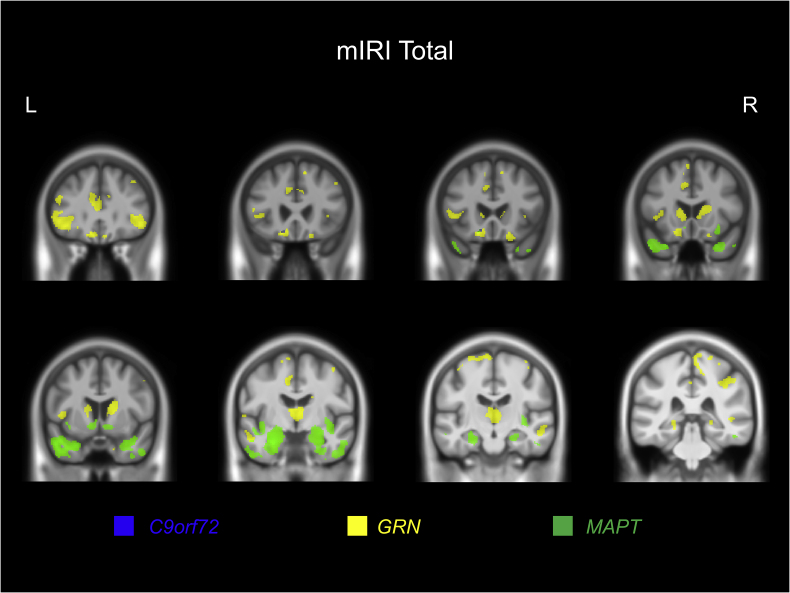
Neural correlates of the modified Interpersonal Reactivity Index (mIRI) Total score. Results for all three genetic groups are displayed at p < .001 uncorrected. A study-specific T1-weighted MRI template in MNI space was used to show results. Green represents the *MAPT* group, yellow for the *GRN* group, and blue for the *C9orf72* group.

For the *C9orf72* expansion carriers, although no significant results were found after multiple corrections, at an uncorrected p < .001, the mIRI Total score was positively correlated with left orbitofrontal cortex volume (see Table S7 and Fig. 3).

For *GRN* mutation carriers, the mIRI Total scores were positively associated with left frontal lobe volume (specifically, superior, middle and orbitofrontal gyri) at p < .05 corrected for multiple comparisons. At the less strict significance level of p < .001 uncorrected, more widespread involvement was seen including bilateral involvement of the frontal lobe (superior, middle and inferior frontal gyri, and orbitofrontal cortex), anterior cingulate, insula, temporal lobe, parietal lobe (including precuneus), and subcortical structures (caudate and thalamus) in particular (see Table S7 and Fig. 3).

In *MAPT* mutation carriers, a positive relationship between mIRI Total scores and grey matter volume was seen bilaterally in the temporal cortex (particularly the entorhinal area and temporal pole), and the medial temporal lobe (hippocampus and amygdala) as well as the insula and orbitofrontal lobe bilaterally after correction for multiple comparisons. At the less strict significance level of p < .001 uncorrected, an association with basal ganglia (particularly nucleus accumbens) was also seen (see Table S7 and Fig. 3).

A very similar set of neuroanatomical associations were seen in each genetic group when correlating the mIRI PT subscore with grey matter volume (Table S8 and Figure S2). However, for the EC subscore there were no significant findings at p < .05 corrected for multiple comparisons, but at an uncorrected p < .001, both the *GRN* and *MAPT* groups had a relatively similar network of associated regions albeit to a lesser extent than in the Total score or PT subscore analysis (Table S9 and Figure S2). In the *C9orf72* group, as well as a similar association with the left orbitofrontal cortex as seen in the other analyses, there was also a correlation with bilateral insula and putamen volume.

## 4 Discussion

In the present study, we have demonstrated that the mIRI detects empathy deficits in fully symptomatic (CDR 1+) familial FTD, as well as in very mildly symptomatic/prodromal (CDR .5) *C9orf72* expansion carriers, although it does not distinguish impaired empathy when comparing other prodromal or asymptomatic individuals. Neural correlates of empathy varied between genetic groups: scores were associated with the left orbitofrontal cortex in all three groups, but additionally more widespread cortical and subcortical regions for *GRN* mutation carriers, and the anteromedial temporal lobe and insula for *MAPT* mutation carriers.

Investigation of mutation negative healthy controls in the GENFI cohort has enabled study of the mIRI in a large group of healthy individuals, generating normative data which can be utilised in future research. We found there to be no effect of age or years spent in education on mIRI Total scores. By contrast, a significant effect of sex was observed, with females scoring higher than their male counterparts, a finding consistently described across the empathy literature (Christov-Moore et al., 2014; Hoffmann, Kessler, Eppe, Rukavin & Traue, 2010; Rueckert & Naybar, 2008), and, more specifically, on all subscales of the IRI (Chrysikou & Thompson, 2016; Davis, 1980; De Corte et al., 2007; Hawk et al., 2013).

Fully symptomatic mutation carriers in all genetic groups scored worse on all three measures of empathy than controls, as well as when compared with their prodromal and asymptomatic counterparts. Such a finding is consistent with previous work in sporadic FTD, in which empathy is significantly reduced in patients relative to controls (Baez et al., 2014; Hazelton et al., 2017; Multani et al., 2019; Rankin et al., 2005, 2006; Shany-Ur et al., 2012). Results indicate that the mIRI is effective at detecting empathy-based behavioural changes during the symptomatic period of FTD.

Reduced empathy is more commonly observed in *C9orf72* mutation carriers, relative to other genetic mutations (Woollacott & Rohrer, 2016). Notably in this study, very mildly symptomatic/prodromal *C9orf72* expansion carriers scored significantly lower than controls on the mIRI Total score. Similar findings of social cognitive deficits have been observed in a recent GENFI study (Russell et al., 2020), in which presymptomatic *C9orf72* mutation carriers in proximity to symptom onset were observed to have impairment of facial emotion recognition. The question remains as to whether empathy deficits only present in later stages of disease progression (after full symptom onset in *GRN* and *MAPT* mutations and during the prodromal stage in *C9orf72* expansions) or whether the mIRI is an insensitive psychometric measure of such changes. Future work employing novel social cognitive tasks in presymptomatic FTD cohorts is essential to uncouple these possibilities.

Although altered social conduct and personality changes manifest in PPA and FTD-ALS (Calabria et al., 2009; Fittipaldi et al., 2019; Gorno-Tempini et al., 2011; Hazelton et al., 2017; Hsieh et al.,2013; Kumfor et al., 2016; Rankin et al., 2005; Savage et al., 2014; Strong et al., 2009), the syndromes are predominantly characterised by progressive language and speech difficulties, or by impaired motor functioning, respectively (Calabria et al., 2009; Gorno-Tempini et al., 2011; Hazelton et al., 2017; Woollacott & Rohrer, 2016). The empathy literature for FTD-ALS is limited, whilst inconsistent findings have been reported in regards to which components of empathy are affected in PPA, e.g., Rankin et al. (2005) noted a diminished capacity for empathy in both cognitive and affective facets of empathy, whilst Calabria and colleagues (2009) (Calabria et al., 2009) found only cognitive domains were impacted. In contrast, studies investigating bvFTD have consistently reported impaired empathy (Baez et al., 2014; Eslinger et al., 2011; Fernandez-Duque et al., 2010; Shany-Ur et al., 2012; Woollacott & Rohrer, 2016) with such deficits forming a core aspect of the diagnostic criteria for this condition (Baez et al., 2014; Cerami et al., 2014; Rascovsky et al., 2011; Strong et al., 2009). Our study adds to this literature, revealing that bvFTD participants scored significantly lower on the mIRI relative to the PPA phenotype group and controls, as has been previously described (Eslinger et al., 2011; Rankin et al., 2006). Notably, however, the FTD-ALS group also scored significantly lower on the mIRI relative to PPA participants, whilst both PPA and FTD-ALS groups also scored comparatively worse than controls on each component measure of empathy, therefore contributing to the limited FTD-ALS empathy literature and supporting the previous findings of Rankin et al. (2005).

Empathy was observed to decrease with increasing disease severity (as determined by scores on the CDR® plus NACC FTLD-SB) - a finding previously described in people within the FTD spectrum (Hsieh et al., 2013). As progressive regional atrophy correlates with increasing disease severity (Seeley et al., 2008), it is to be expected that symptoms become increasingly prominent with deterioration of the disease (Irish, Kumfor, Hodges & Piguet, 2013). Our finding is therefore supportive of prior research.

Impairment on tasks of empathy are likely to involve the breakdown of a number of processes within the brain. Consistent with this, a network of regions was found to be associated with mIRI Total score in the present study, with distinct but overlapping regions in the different genetic groups.

The one region that overlapped in the VBM findings of all three groups was the orbitofrontal cortex, a region that evaluates the reward-value and punishment-potential of a stimulus (Rankin et al., 2006; Rudebeck et al., 2008), and by extension therefore plays an important role in empathic responsivity, through interpretation of stimulus emotional salience (Fittipaldi et al., 2019; Kumfor et al., 2016; Shany-Ur et al., 2012). This association of orbitofrontal cortex degeneration and defective empathy has been previously described in healthy controls (Völlm et al., 2006) as well as in people with sporadic FTD (Rankin & et al., 2005, 2006).

In the *GRN* mutation carriers, neural correlates were more widespread including the frontal cortex, anterior insula and anterior cingulate, namely the salience network. This neural network plays an important role in social cognition through allocation of attentional resources upon detection of emotionally salient stimuli (Farb et al., 2013; Pasquini et al., 2020; Wittenberg et al., 2008). Integrity of this network has been implicated with social-emotional functioning in healthy controls (Shamay-Tsoory, 2011), as well as in clinical subpopulations (Menon & Uddin, 2010). Importantly, the neural network is selectively degenerated in FTD (Cash et al., 2018; Lough et al., 2006; Pasquini et al., 2020; Rohrer et al., 2015; Seeley et al., 2008; Shany-Ur et al., 2012; Viskontas et al., 2007), likely underlying the diseases’ characteristic behavioural disturbances.

The anterior cingulate has been associated with memory retrieval, which is critical for attentional processes (Fink et al., 1996; Maguire & Mummery, 1999), contributes to empathy to pain as a component of the pain matrix (Decety, Echols & Correll, 2010; Shamay-Tsoory, 2011), and is suggested to facilitate motor response to salient stimuli due to functional connectivity with motor areas (Menon & Uddin, 2010; Rudebeck et al., 2008). Critically, grey matter volume of the cingulate has been directly linked to ratings on empathy tasks previously (McCreary, Marchant & Davis, 2018;; Rankin et al., 2006; Shamay-Tsoory, 2011) and was correlated with *GRN* mutation carriers’ mIRI scores in the present study.

The insula is a functionally heterogeneous brain region, responsible for autonomic regulation and somatosensory processing (Menon & Uddin, 2010). It mediates emotion comprehension and expression, particularly for stimuli with a negative valence (Lough et al., 2006; Rankin et al., 2006; Singer et al., 2004; Singer, 2006), through the integration of somatosensory experience (anterior insula) with homeostatic signals and physiological states (posterior insula) and recruitment of attentional resources (Hazelton et al., 2017; Menon & Uddin, 2010). A subjective awareness of one's emotional state is produced in this process, which in turn may heighten affective empathic response (Carr et al., 2003; Shdo et al., 2018). Critically, this region is one of the earliest to be affected in genetic FTD patients, with cortical atrophy observed around 10 years prior to symptom onset (Cash et al., 2018; Rohrer & et al., 2015; Seeley et al., 2008), suggesting this region is an early pathological target in FTD. Supporting this, in our study, involvement of the insula was associated with both *GRN* and *MAPT* mutation carriers' mIRI scores as well as the *C9orf7*2 EC subscore. Together, the fronto-insula network is responsible for processing socially significant cues (Shany-Ur et al., 2012).

Regions specifically associated with *MAPT* mutation carriers’ scores included the hippocampus, amygdala, and entorhinal area. These structures form part of the limbic system and are subsequently critical in generating and processing emotions (Carr, Iacoboni, Dubeau, Mazziotta & Lenzi, 2003; Shdo et al., 2018). Notably, fronto-limbic structures have been implicated in the lower scores of people with bvFTD on the PT and EC subscales (Eslinger et al., 2011) and, more specifically, the amygdala was found to be responsible for discrete emotion processing in sporadic FTD (Perry et al., 2001). Grey matter volume of the entorhinal area has been linked with affect sharing (McCreary et al., 2018) and the hippocampus mediates autobiographical memory retrieval. The anatomical findings of the present study are therefore consistent with the previous research evaluating the neural correlates of empathy and provide support for the role of such structures in mediating empathic abilities of genetic FTD patients.

The temporal poles were also associated with *MAPT* mutation carriers' scores. They are described as multimodal epicentres that integrate sensory information with limbic inputs to form personalized representations of emotional input (Harada et al., 2009; Rankin et al., 2006; Uchiyama et al., 2006). The left temporal pole is involved in linguistic processes such as contriving sentence meaning (Vandenberghe, Nobre & Price, 2002), as well as autobiographical memory retrieval tasks (Maguire, Mummery & Büchel, 2000; Singer et al., 2004; Singer, 2006). Lesions to the left temporal pole are associated with an impaired ability to comprehend lies (Harada et al., 2009) and sarcasm (Uchiyama et al., 2006), and poor performance on theory of mind (Lough et al., 2006) and emotion attribution tasks (Cerami et al., 2014). Considering this, Frith and Frith (2003) described the region's role in ‘script’ retrieval; scripts utilise semantic and emotional information of repeated experiences to predict the likely sequence of events in any given situation. By extension, scripts allow for inferences of others' likely behaviours, intentions or goals to be made (Schank & Abelson, 1977), all of which contribute to our ability to mentalize. Mentalizing forms an important component of empathy as it enables an understanding of others' mental states and perceptions, following self-other distinction (Pasquini et al., 2020). Script retrieval aids this process of mentalizing by providing a situational framework within which the social cognitive process can be applied (Frith & Frith, 2003; Uchiyama et al., 2006). The role played by the temporal poles in empathy is confirmed by previous observations of bilateral involvement in healthy controls (Decety et al., 2010; Menon & Uddin, 2010) and right temporal pole correlations in people with FTD (Rankin et al., 2006). Findings of our study are consistent with prior research, as grey matter volume of the temporal poles were associated with *MAPT* mutation carriers' scores particularly. By mediating script recruitment, past experiences can be utilised to understand salient experiences happening to the self and to others, all of which is required to produce empathic understanding.

The putamen was also observed to be associated with scores in the *GRN* and *MAPT* groups as well as the EC subscore in the *C9orf72* group - a region implicated in limbic connectivity, dysfunction of which contributes to defective behaviour in sporadic FTD (Farb et al., 2013). Interestingly, given the findings in the *C9orf72* expansion carriers the putamen has previously been linked with EC ratings of people with neurodegenerative disease, including sporadic FTD (McCreary et al., 2018) i.e., may be a substrate for affective empathy. Also implicated in theory of mind and emotion recognition functioning is the caudate (Russell et al., 2020) - a region observed to be associated with *GRN* and *MAPT* mutation carriers’ empathy ratings in our study. Support for this finding comes from Rankin and colleagues (2006) (Rankin et al., 2006) who observed that right caudate volume was correlated with total empathy of people with sporadic FTD, and Shdo and colleagues (2018) (McCreary et al., 2018) who implicated the region with prosocial motivation, recognised as an affective component of empathy.

Limbic connectivity of the thalamus has also been attributed to aberrant behaviours of sporadic FTD patients (Farb et al., 2013) and was bilaterally correlated with *GRN* mutation carriers’ empathy scores in the present study. In addition, the region has previously been implicated in emotion processing and theory of mind tasks of *GRN* mutation carriers (Russell et al., 2020) and forms part of the salience network (Wittenberg et al., 2008).

Distinguishing groups based by genetic mutation as opposed to clinical presentation or predominant focal atrophy patterns may account for these novel neuroanatomical findings as this methodological approach has not formerly been employed. As previously described, each mutation type produces distinctive, gene-specific degeneration. Future work is required, comparing sporadic and genetic patients with equivalent diagnoses, to understand and identify any differences between groups. Moreover, pathological phenotypes are recognised for each gene, however, clinical presentation is imperfect and variable (Lashley et al., 2015). Genetic testing, as used in the present study, ensures certainty regarding clinical diagnosis of FTD, though the same cannot be assumed in sporadic cases. Drawing comparisons between sporadic and genetic FTD patients should therefore be performed with caution. Finally, a large sample size was utilised in the present study. Prior to this, examined cohorts have been smaller, possibly contributing to the disparity in results.

When considering the affective and cognitive components of empathy, it has been theorized that distinct neuroanatomical regions are responsible for mediating each construct (Christov-Moore et al., 2014; Fink et al., 1996 Hazelton et al., 2017; Pasquini et al., 2020; ), though FTD research has been inconclusive about such a proposal. Rankin and colleagues (2006) (Rankin et al., 2006) found significant overlap between the neural correlates associated with EC and PT components of empathy, with only trends of differences. In contrast, Eslinger and colleagues (2011) (Eslinger et al., 2011) observed distinct neural correlates mediating each component. In the present study, no major differences were observed between the PT and EC subscales and their associated clusters, as identified by VBM analysis, between familial groups except in the *C9orf72* group where additional insula and putamen involvement was seen. A possible explanation for such a finding comes from Rankin and colleagues’ (2006) (Rankin et al., 2006) proposal of overlapping anatomical regions as a result of highly correlated subscale scores. Following this, it is possible the same is true in our study. A second interpretation comes from McCreary et al. (2018) (Shdo et al., 2018) who suggested that a suitable empathic response is mediated by balanced, simultaneous activation of both component networks of empathy and, by extension, similar anatomical areas are engaged. Therefore, though separate brain regions underpin the respective components of empathy, rendering the networks dissociable, social experiences requiring an empathic response are likely to activate and evoke both (Fink et al., 1996; Pasquini et al., 2020). These theories provide support for our study by elucidating how the identified neural networks are engaged in real-life empathic experiences, following the logic that both are fundamental in mediating this process.

The first limitation of the study is the use of a caregiver-report questionnaire. Though observer-based measures are more ecologically valid and have yielded valuable data previously (Rankin et al., 2005), they are nevertheless limited by their dependence on informants varying reliability (Shany-Ur et al., 2012). Despite this, they have an advantage of capturing real-life empathic behaviour, independent of patients’ anosognosia (Fittipaldi et al., 2019; McCreary et al., 2018) and social desirability effects (Menon & Uddin, 2010). Furthermore, the measure is considered reliable, reasonably easy to complete and reproducible (Bernhardt & Singer, 2012). Secondly, although a large cohort of participants were recruited, once stratified there were relatively small numbers in some of the groups. To overcome this issue, further data collection as part of GENFI and similar studies is required. Lastly, the majority of patients with the PPA phenotype had the nonfluent variant and therefore it was not possible to analyse PPA subtypes further in the current study.

Going forward, refinement of the mIRI or development of novel social cognition empathy tasks that are sensitive to presymptomatic changes is essential. The assessment of empathy deficits in presymptomatic clinical populations is a prerequisite to a comprehensive picture of symptom progression and disease trajectory of FTD. Additionally required is a future longitudinal study, assessing the progressive changes in empathy and its neural substrates in people with familial FTD, particularly in individuals who phenoconvert, in order to further the current understanding of emerging deficits and their evolution. Such knowledge is beneficial for researchers as it will enable the identification of individuals suitable for clinical trials, indicate the appropriate time to implement therapies, and allow patient response to such therapies to be tracked (Greaves & Rohrer, 2019; Rohrer et al., 2013; Rohrer et al., 2015), but also for caregivers and family members of at-risk individuals. Defective empathy is a highly burdensome symptom of FTD (Fittipaldi et al., 2019; Savage et al., 2014; Snowden, 2018), exacerbated by patients’ anosognosia for their declining emotional responsivity, which renders them unaware of their behavioural changes (Englund et al., 1994; Eslinger et al., 2005; Eslinger et al., 2011; Rankin et al., 2006; Shany-Ur et al., 2012; Viskontas et al., 2007; Woollacott & Rohrer, 2016). Accordingly, caregiver distress is reportedly much higher in FTD, relative to other neurodegenerative diseases (Hazelton et al., 2017; Hsieh et al., 2013; Warren et al., 2013). Greater comprehension of clinical trajectory and the presymptomatic stages of disease may therefore help to reduce caregiver burden by ensuring better environmental management and forward planning can be implemented, focused on symptom alleviation (Völlm et al., 2006), whilst simultaneously furthering the field of research.

## 5 Conclusion

We have provided clear evidence of impaired empathy in symptomatic participants in all three genetic groups, as well as prodromal *C9orf72* expansion carriers, finding that empathic abilities decrease with increasing disease severity. Additionally, we have demonstrated that mutation-specific neurodegeneration is correlated with informant ratings of participant empathy. Our results delineate the neural correlates of empathy in genetic FTD, emphasising the role played by the orbitofrontal lobe. Together, these findings contribute to the current understanding of this complex, multifaceted construct, foundational to human social-emotional interaction. We conclude that whilst the mIRI is beneficial for the study of symptomatic FTD participants, its use in future clinical trials targeting presymptomatic individuals may be of limited value. More sensitive measures and a greater understanding of longitudinal changes in empathy over time are therefore vital next steps.

## CRediT author statement

Phoebe Foster: Conceptualization (supporting); Formal analysis (lead); Visualization (lead); Writing - Original Draft (lead); Writing - Review & Editing (equal). Lucy L. Russell: Conceptualization (supporting); Formal analysis (supporting); Visualization (supporting); Writing - Review & Editing (equal). Georgia Peakman: Data Curation (lead); Investigation (equal); Writing - Review & Editing (equal). Rhian S. Convery: Data Curation (lead); Investigation (equal); Writing - Review & Editing (equal). Arabella Bouzigues: Data Curation (lead); Investigation (equal); Writing - Review & Editing (equal). Caroline V. Greaves: Project administration (equal); Resources (supporting); Investigation (equal). Martina Bocchetta: Formal analysis (supporting); Writing - Review & Editing (equal). David M. Cash: Formal analysis (supporting); Writing - Review & Editing (equal). Jennifer Nicholas: Formal analysis (supporting); Writing - Review & Editing (equal). John van Swieten: Resources (equal); Project administration (equal); Funding acquisition (equal); Investigation (supporting); Writing - Review & Editing (supporting). Lize Jiskoot: Resources (equal); Project administration (equal); Funding acquisition (equal); Investigation (supporting); Writing - Review & Editing (supporting). Fermin Moreno: Resources (equal); Project administration (equal); Funding acquisition (equal); Investigation (supporting); Writing - Review & Editing (supporting). Raquel Sanchez-Valle: Resources (equal); Project administration (equal); Funding acquisition (equal); Investigation (supporting); Writing - Review & Editing (supporting). Robert Laforce: Resources (equal); Project administration (equal); Funding acquisition (equal); Investigation (supporting); Writing - Review & Editing (supporting). Caroline Graff: Resources (equal); Project administration (equal); Funding acquisition (equal); Investigation (supporting); Writing - Review & Editing (supporting). Mario Masellis: Resources (equal); Project administration (equal); Funding acquisition (equal); Investigation (supporting); Writing - Review & Editing (supporting). Maria Carmela Tartaglia: Resources (equal); Project administration (equal); Funding acquisition (equal); Investigation (supporting); Writing - Review & Editing (supporting). James B Rowe: Resources (equal); Project administration (equal); Funding acquisition (equal); Investigation (supporting); Writing - Review & Editing (supporting). Barbara Borroni: Resources (equal); Project administration (equal); Funding acquisition (equal); Investigation (supporting); Writing - Review & Editing (supporting). Elizabeth Finger: Resources (equal); Project administration (equal); Funding acquisition (equal); Investigation (supporting); Writing - Review & Editing (supporting). Matthis Synofzik: Resources (equal); Project administration (equal); Funding acquisition (equal); Investigation (supporting); Writing - Review & Editing (supporting). Daniela Galimberti: Resources (equal); Project administration (equal); Funding acquisition (equal); Investigation (supporting); Writing - Review & Editing (supporting). Rik Vandenberghe: Resources (equal); Project administration (equal); Funding acquisition (equal); Investigation (supporting); Writing - Review & Editing (supporting). Alexandre de Mendonça: Resources (equal); Project administration (equal); Funding acquisition (equal); Investigation (supporting); Writing - Review & Editing (supporting). Chris Butler: Resources (equal); Project administration (equal); Funding acquisition (equal); Investigation (supporting); Writing - Review & Editing (supporting). Alex Gerhard: Re- sources (equal); Project administration (equal); Funding acquisition (equal); Investigation (supporting); Writing - Review & Editing (supporting). Simon Ducharme: Resources (equal); Project administration (equal); Funding acquisition (equal); Investigation (supporting); Writing - Review & Editing (supporting). Isabelle Le Ber: Resources (equal); Project administration (equal); Funding acquisition (equal); Investigation (supporting); Writing - Re-view & Editing (supporting). Fabrizio Tagliavini: Resources (equal); Project administration (equal); Funding acquisition (equal); Investigation (supporting); Writing - Review & Editing (supporting). Isabel Santana: Resources (equal); Project administration (equal); Funding acquisition (equal); Investigation (supporting); Writing - Review & Editing (supporting). Florence Pasquier: Resources (equal); Project administration (equal); Funding acquisition (equal); Investigation (supporting); Writing - Re-view & Editing (supporting). Johannes Levin: Resources (equal); Project administration (equal); Funding acquisition (equal); Investigation (supporting); Writing - Review & Editing (supporting). Adrian Danek: Resources (equal); Project administration (equal); Funding acquisition (equal); Investigation (supporting); Writing - Review & Editing (supporting). Markus Otto: Resources (equal); Project administration (equal); Funding acquisition (equal); Investigation (supporting); Writing - Review & Editing (supporting). Sandro Sorbi: Resources (equal); Project administration (equal); Funding acquisition (equal); Investigation (supporting); Writing - Review & Editing (supporting). Jonathan D Rohrer: Conceptualization (lead); Supervision (lead); Formal analysis (supporting); Writing - Review & Editing (equal); Project administration (equal); Funding acquisition (lead).

## Open practices

The study in this article earned an Open Materials badge for transparent practices. Study data has not been deposited due to ethics committee restrictions, but data is available to researchers on reasonable request to genfi@ucl.ac.uk.

We report how we determined our sample size, all data exclusions, all inclusion/exclusion criteria, whether inclusion/exclusion criteria were established prior to data analysis, all manipulations, and all measures in the study. The conditions of our ethics approval do not permit public archiving of anonymised study data. Readers seeking access to the data should contact the lead author Professor Jonathan Rohrer at UCL (genfi@ucl.ac.uk). Access will be granted to named individuals in accordance with ethical procedures governing the reuse of sensitive data. Specifically, requestors must complete a formal data sharing agreement. Statistical analysis code is available here: https://osf.io/u9bdm/. No part of the study procedures or analyses were pre-registered prior to the research being conducted.

## Declarations of competing interest

None.

## Appendix

### GENFI Consortium Authors

• Sónia Afonso - Instituto Ciencias Nucleares Aplicadas a Saude, Universidade de Coimbra, Coimbra, Portugal;
• Maria Rosario Almeida - Faculty of Medicine, University of Coimbra, Coimbra, Portugal;
• Sarah Anderl-Straub – Department of Neurology, University of Ulm, Ulm, Germany;
• Christin Andersson - Department of Clinical Neuroscience, Karolinska Institutet, Stockholm, Sweden;
• Anna Antonell - Alzheimer's disease and Other Cognitive Disorders Unit, Neurology Service, Hospital Clínic, Barcelona, Spain;
• Silvana Archetti - Biotechnology Laboratory, Department of Diagnostics, ASST Brescia Hospital, Brescia, Italy;
• Andrea Arighi - Fondazione IRCCS Ca’ Granda Ospedale Maggiore Policlinico, Neurodegenerative Diseases Unit, Milan, Italy; University of Milan, Centro Dino Ferrari, Milan, Italy;
• Mircea Balasa - Alzheimer's disease and Other Cognitive Disorders Unit, Neurology Service, Hospital Clínic, Barcelona, Spain;
• Myriam Barandiaran - Cognitive Disorders Unit, Department of Neurology, Donostia University Hospital, San Sebastian, Gipuzkoa, Spain; Neuroscience Area, Biodonostia Health Research Institute, San Sebastian, Gipuzkoa, Spain;
• Nuria Bargalló - Imaging Diagnostic Center, Hospital Clínic, Barcelona, Spain;
• Robart Bartha - Department of Medical Biophysics, The University of Western Ontario, London, Ontario, Canada; Centre for Functional and Metabolic Mapping, Robarts Research Institute, The University of Western Ontario, London, Ontario, Canada;
• Benjamin Bender - Department of Diagnostic and Interventional Neuroradiology, University of Tübingen, Tübingen, Germany;
• Alberto Benussi - Centre for Neurodegenerative Disorders, Department of Clinical and Experimental Sciences, University of Brescia, Italy;
• Maxime Bertoux – Inserm 1172, Lille, France; CHU, CNR-MAJ, Labex Distalz, LiCEND Lille, France;
• Anne Bertrand - Sorbonne Université, Paris Brain Institute – Institut du Cerveau – ICM, Inserm U1127, CNRS UMR 7225, AP-HP - Hôpital Pitié-Salpêtrière, Paris, France; Inria, Aramis project-team, F-75013, Paris, France; Centre pour l'Acquisition et le Traitement des Images, Institut du Cerveau et la Moelle, Paris, France
• Valentina Bessi - Department of Neuroscience, Psychology, Drug Research, and Child Health, University of Florence, Florence, Italy;
• Sandra Black - Sunnybrook Health Sciences Centre, Sunnybrook Research Institute, University of Toronto, Toronto, Canada;
• Sergi Borrego-Ecija - Alzheimer's disease and Other Cognitive Disorders Unit, Neurology Service, Hospital Clínic, Barcelona, Spain;
• Jose Bras - Center for Neurodegenerative Science, Van Andel Institute, Grand Rapids, Michigan, MI 49503, USA;
• Alexis Brice - Sorbonne Université, Paris Brain Institute – Institut du Cerveau – ICM, Inserm U1127, CNRS UMR 7225, AP-HP - Hôpital Pitié-Salpêtrière, Paris, France; Reference Network for Rare Neurological Diseases (ERN-RND);
• Rose Bruffaerts - Laboratory for Cognitive Neurology, Department of Neurosciences, KU Leuven, Leuven, Belgium;
• Agnès Camuzat - Sorbonne Université, Paris Brain Institute – Institut du Cerveau – ICM, Inserm U1127, CNRS UMR 7225, AP-HP - Hôpital Pitié-Salpêtrière, Paris, France;
• Marta Cañada - CITA Alzheimer, San Sebastian, Gipuzkoa, Spain
• Valentina Cantoni - Centre for Neurodegenerative Disorders, Neurology Unit, Department of Clinical and Experimental Sciences, University of Brescia, Brescia, Italy;
• Paola Caroppo - Fondazione IRCCS Istituto Neurologico Carlo Besta, Milano, Italy;
• David Cash – Dementia Research Centre, Department of Neurodegenerative Disease, UCL Institute of Neurology, Queen Square, London, UK;
• Miguel Castelo-Branco - Faculty of Medicine, University of Coimbra, Coimbra, Portugal;
• Olivier Colliot - Sorbonne Université, Paris Brain Institute – Institut du Cerveau – ICM, Inserm U1127, CNRS UMR 7225, AP-HP - Hôpital Pitié-Salpêtrière, Paris, France; Inria, Aramis project-team, F-75013, Paris, France; Centre pour l'Acquisition et le Traitement des Images, Institut du Cerveau et la Moelle, Paris, France;
• Thomas Cope – Department of Clinical Neuroscience, University of Cambridge, Cambridge, UK;
• Vincent Deramecourt - Univ Lille, France; Inserm 1172, Lille, France; CHU, CNR-MAJ, Labex Distalz, LiCEND Lille, France;
• María de Arriba - Neuroscience Area, Biodonostia Health Research Institute, San Sebastian, Gipuzkoa, Spain;
• Giuseppe Di Fede - Fondazione IRCCS Istituto Neurologico Carlo Besta, Milano, Italy;
• Alina Díez - Neuroscience Area, Biodonostia Health Research Institute, San Sebastian, Gipuzkoa, Spain
• Diana Duro - Faculty of Medicine, University of Coimbra, Coimbra, Portugal;
• Chiara Fenoglio - Fondazione IRCCS Ca’ Granda Ospedale Maggiore Policlinico, Neurodegenerative Diseases Unit, Milan, Italy; University of Milan, Centro Dino Ferrari, Milan, Italy;
• Camilla Ferrari - Department of Neuroscience, Psychology, Drug Research, and Child Health, University of Florence, Florence, Italy;
• Catarina B. Ferreira -Laboratory of Neurosciences, Institute of Molecular Medicine, Faculty of Medicine, University of Lisbon, Lisbon, Portugal;
• Nick Fox – Dementia Research Centre, Department of Neurodegenerative Disease, UCL Institute of Neurology, Queen Square, London, UK;
• Morris Freedman - Baycrest Health Sciences, Rotman Research Institute, University of Toronto, Toronto, Canada;
• Giorgio Fumagalli - Fondazione IRCCS Ca’ Granda Ospedale Maggiore Policlinico, Neurodegenerative Diseases Unit, Milan, Italy; University of Milan, Centro Dino Ferrari, Milan, Italy;
• Aurélie Funkiewiez - Centre de référence des démences rares ou précoces, IM2A, Département de Neurologie, AP-HP - Hôpital Pitié-Salpêtrière, Paris, France; Sorbonne Université, Paris Brain Institute – Institut du Cerveau – ICM, Inserm U1127, CNRS UMR 7225, AP-HP - Hôpital Pitié-Salpêtrière, Paris, France;
• Alazne Gabilondo -Neuroscience Area, Biodonostia Health Research Institute, San Sebastian, Gipuzkoa, Spain;
• Roberto Gasparotti - Neuroradiology Unit, University of Brescia, Brescia, Italy
• Serge Gauthier - Alzheimer Disease Research Unit, McGill Centre for Studies in Aging, Department of Neurology & Neurosurgery, McGill University, Montreal, Québec, Canada;
• Stefano Gazzina - Neurology, ASST Brescia Hospital, Brescia, Italy
• Giorgio Giaccone - Fondazione IRCCS Istituto Neurologico Carlo Besta, Milano, Italy;
• Ana Gorostidi - Neuroscience Area, Biodonostia Health Research Institute, San Sebastian, Gipuzkoa, Spain;
• Caroline Greaves – Dementia Research Centre, Department of Neurodegenerative Disease, UCL Institute of Neurology, Queen Square, London, UK;
• Rita Guerreiro - Center for Neurodegenerative Science, Van Andel Institute, Grand Rapids, Michigan, MI 49503, USA;
• Carolin Heller – Dementia Research Centre, Department of Neurodegenerative Disease, UCL Institute of Neurology, Queen Square, London, UK;
• Tobias Hoegen - Neurologische Klinik, Ludwig-Maximilians-Universität München, Munich, Germany;
• Begoña Indakoetxea - Cognitive Disorders Unit, Department of Neurology, Donostia University Hospital, San Sebastian, Gipuzkoa, Spain; Neuroscience Area, Biodonostia Health Research Institute, San Sebastian, Gipuzkoa, Spain;
• Vesna Jelic - Division of Clinical Geriatrics, Karolinska Institutet, Stockholm, Sweden;
• Hans-Otto Karnath - Division of Neuropsychology, Hertie-Institute for Clinical Brain Research and Center of Neurology, University of Tübingen, Tübingen, Germany;
• Ron Keren -The University Health Network, Toronto Rehabilitation Institute, Toronto, Canada;
• Gregory Kuchcinski - Univ Lille, France; Inserm 1172, Lille, France; CHU, CNR-MAJ, Labex Distalz, LiCEND Lille, France;
• Tobias Langheinrich - Division of Neuroscience and Experimental Psychology, Wolfson Molecular Imaging Centre, University of Manchester, Manchester, UK; Manchester Centre for Clinical Neurosciences, Department of Neurology, Salford Royal NHS Foundation Trust, Manchester, UK;
• Thibaud Lebouvier - Univ Lille, France; Inserm 1172, Lille, France; CHU, CNR-MAJ, Labex Distalz, LiCEND Lille, France;
• Maria João Leitão - Centre of Neurosciences and Cell Biology, Universidade de Coimbra, Coimbra, Portugal;
• Albert Lladó - Alzheimer's disease and Other Cognitive Disorders Unit, Neurology Service, Hospital Clínic, Barcelona, Spain;
• Gemma Lombardi - Department of Neuroscience, Psychology, Drug Research and Child Health, University of Florence, Florence, Italy;
• Sandra Loosli -Neurologische Klinik, Ludwig-Maximilians-Universität München, Munich, Germany;
• Carolina Maruta - Laboratory of Language Research, Centro de Estudos Egas Moniz, Faculty of Medicine, University of Lisbon, Lisbon, Portugal;
• Simon Mead - MRC Prion Unit, Department of Neurodegenerative Disease, UCL Institute of Neurology, Queen Square, London, UK;
• Lieke Meeter - Department of Neurology, Erasmus Medical Center, Rotterdam, Netherlands;
• Gabriel Miltenberger - Faculty of Medicine, University of Lisbon, Lisbon, Portugal;
• Rick van Minkelen - Department of Clinical Genetics, Erasmus Medical Center, Rotterdam, Netherlands;
• Sara Mitchell - Sunnybrook Health Sciences Centre, Sunnybrook Research Institute, University of Toronto, Toronto, Canada;
• Katrina Moore – Dementia Research Centre, Department of Neurodegenerative Disease, UCL Institute of Neurology, Queen Square, London UK;
• Benedetta Nacmias - Department of Neuroscience, Psychology, Drug Research and Child Health, University of Florence, Florence, Italy;
• Annabel Nelson - Dementia Research Centre, Department of Neurodegenerative Disease, UCL Institute of Neurology, Queen Square, London, UK;
• Linn Öijerstedt - Center for Alzheimer Research, Division of Neurogeriatrics, Department of Neurobiology, Care Sciences and Society, Bioclinicum, Karolinska Institutet, Solna, Sweden; Unit for Hereditary Dementias, Theme Aging, Karolinska University Hospital, Solna, Sweden;
• Jaume Olives - Alzheimer's disease and Other Cognitive Disorders Unit, Neurology Service, Hospital Clínic, Barcelona, Spain;
• Sebastien Ourselin - School of Biomedical Engineering & Imaging Sciences, King's College London, London, UK;
• Alessandro Padovani - Centre for Neurodegenerative Disorders, Department of Clinical and Experimental Sciences, University of Brescia, Italy
• Jessica Panman – Department of Neurology, Erasmus Medical Center, Rotterdam, Netherlands;
• Janne M. Papma - Department of Neurology, Erasmus Medical Center, Rotterdam;
• Yolande Pijnenburg - Amsterdam University Medical Centre, Amsterdam VUmc, Amsterdam, Netherlands;
• Cristina Polito - Department of Biomedical, Experimental and Clinical Sciences “Mario Serio”, Nuclear Medicine Unit, University of Florence, Florence, Italy
• Enrico Premi - Stroke Unit, ASST Brescia Hospital, Brescia, Italy
• Sara Prioni - Fondazione IRCCS Istituto Neurologico Carlo Besta, Milano, Italy;
• Catharina Prix -Neurologische Klinik, Ludwig-Maximilians-Universität München, Munich, Germany;
• Rosa Rademakers [as London Ontario geneticist] - Department of Neurosciences, Mayo Clinic, Jacksonville, Florida, USA;
• Veronica Redaelli -Fondazione IRCCS Istituto Neurologico Carlo Besta, Milano, Italy;
• Daisy Rinaldi - Centre de référence des démences rares ou précoces, IM2A, Département de Neurologie, AP-HP - Hôpital Pitié-Salpêtrière, Paris, France; Sorbonne Université, Paris Brain Institute – Institut du Cerveau – ICM, Inserm U1127, CNRS UMR 7225, AP-HP - Hôpital Pitié-Salpêtrière, Paris, France; Département de Neurologie, AP-HP - Hôpital Pitié-Salpêtrière, Paris, France; Reference Network for Rare Neurological Diseases (ERN-RND);
• Tim Rittman – Department of Clinical Neurosciences, University of Cambridge, Cambridge, UK;
• Ekaterina Rogaeva -Tanz Centre for Research in Neurodegenerative Diseases, University of Toronto, Toronto, Canada;
• Adeline Rollin - CHU, CNR-MAJ, Labex Distalz, LiCEND Lille, France;
• Pedro Rosa-Neto -Translational Neuroimaging Laboratory, McGill Centre for Studies in Aging, McGill University, Montreal, Québec, Canada;
• Giacomina Rossi - Fondazione IRCCS Istituto Neurologico Carlo Besta, Milano, Italy;
• Martin Rossor – Dementia Research Centre, Department of Neurodegenerative Disease, UCL Institute of Neurology, Queen Square, London, UK;
• Beatriz Santiago - Neurology Department, Centro Hospitalar e Universitario de Coimbra, Coimbra, Portugal;
• Dario Saracino – Sorbonne Université, Paris Brain Institute – Institut du Cerveau – ICM, Inserm U1127, CNRS UMR 7225, AP-HP - Hôpital Pitié-Salpêtrière, Paris, France; Inria, Aramis project-team, F-75013, Paris, France; Centre de référence des démences rares ou précoces, IM2A, Département de Neurologie, AP-HP - Hôpital Pitié-Salpêtrière, Paris, France;
• Sabrina Sayah - Sorbonne Université, Paris Brain Institute – Institut du Cerveau – ICM, Inserm U1127, CNRS UMR 7225, AP-HP - Hôpital Pitié-Salpêtrière, Paris, France;
• Elio Scarpini - Fondazione IRCCS Ca’ Granda Ospedale Maggiore Policlinico, Neurodegenerative Diseases Unit, Milan, Italy; University of Milan, Centro Dino Ferrari, Milan, Italy;
• Sonja Schönecker - Neurologische Klinik, Ludwig-Maximilians-Universität München, Munich, Germany;
• Harro Seelaar - Department of Neurology, Erasmus Medical Centre, Rotterdam, Netherlands;
• Elisa Semler -Department of Neurology, University of Ulm, Ulm;
• Rachelle Shafei – Dementia Research Centre, Department of Neurodegenerative Disease, UCL Institute of Neurology, Queen Square, London, UK;
• Christen Shoesmith - Department of Clinical Neurological Sciences, University of Western Ontario, London, Ontario, Canada;
• Imogen Swift - Department of Neurodegenerative Disease, Dementia Research Centre, UCL Institute of Neurology, Queen Square, London, UK;
• Miguel Tábuas-Pereira - Neurology Department, Centro Hospitalar e Universitario de Coimbra, Coimbra, Portugal;
• Mikel Tainta - Neuroscience Area, Biodonostia Health Research Institute, San Sebastian, Gipuzkoa, Spain;
• Ricardo Taipa - Neuropathology Unit and Department of Neurology, Centro Hospitalar do Porto - Hospital de Santo António, Oporto, Portugal;
• David Tang-Wai -The University Health Network, Krembil Research Institute, Toronto, Canada;
• David L Thomas - Neuroimaging Analysis Centre, Department of Brain Repair and Rehabilitation, UCL Institute of Neurology, Queen Square, London, UK;
• Paul Thompson - Division of Neuroscience and Experimental Psychology, Wolfson Molecular Imaging Centre, University of Manchester, Manchester, UK;
• Hakan Thonberg - Center for Alzheimer Research, Division of Neurogeriatrics, Karolinska Institutet, Stockholm, Sweden;
• Carolyn Timberlake – Department of Clinical Neurosciences, University of Cambridge, Cambridge, UK;
• Pietro Tiraboschi - Fondazione IRCCS Istituto Neurologico Carlo Besta, Milano, Italy;
• Emily Todd - Dementia Research Centre, Department of Neurodegenerative Disease, UCL Institute of Neurology, UK;
• Philip Van Damme - Neurology Service, University Hospitals Leuven, Belgium; Laboratory for Neurobiology, VIB-KU Leuven Centre for Brain Research, Leuven, Belgium;
• Mathieu Vandenbulcke - Geriatric Psychiatry Service, University Hospitals Leuven, Belgium; Neuropsychiatry, Department of Neurosciences, KU Leuven, Leuven, Belgium;
• Michele Veldsman - Nuffield Department of Clinical Neurosciences, Medical Sciences Division, University of Oxford, Oxford, UK;
• Ana Verdelho - Department of Neurosciences and Mental Health, Centro Hospitalar Lisboa Norte - Hospital de Santa Maria & Faculty of Medicine, University of Lisbon, Lisbon, Portugal;
• Jorge Villanua - OSATEK, University of Donostia, San Sebastian, Gipuzkoa, Spain;
• Jason Warren – Dementia Research Centre, Department of Neurodegenerative Disease, UCL Institute of Neurology, Queen Square, London, UK;
• Carlo Wilke - Department of Neurodegenerative Diseases, Hertie-Institute for Clinical Brain Research and Center of Neurology, University of Tübingen, Tübingen, Germany; Center for Neurodegenerative Diseases (DZNE), Tübingen, Germany;
• Ione Woollacott – Dementia Research Centre, Department of Neurodegenerative Disease, UCL Institute of Neurology, Queen Square, London, UK;
• Elisabeth Wlasich - Neurologische Klinik, Ludwig-Maximilians-Universität München, Munich, Germany;
• Henrik Zetterberg - Dementia Research Institute, Department of Neurodegenerative Disease, UCL Institute of Neurology, Queen Square, London, UK;
• Miren Zulaica - Neuroscience Area, Biodonostia Health Research Institute, San Sebastian, Gipuzkoa, Spain;
