# Supplementary material for: Examining empathy deficits across familial forms of frontotemporal dementia within the GENFI cohort
Source: Cortex. 2022 May;150:12–28. doi: 10.1016/j.cortex.2022.01.012 (PMC9067453; doi:10.1016/j.cortex.2022.01.012)
Supplement: Multimedia component 1 [file mmc1.docx]

**Supplementary Data**

Table S1: Cumulative frequency of mIRI Total scores in controls. The 5^th^ percentile was used as a cut-off for abnormal scoring such that a score of lower than 38 is considered abnormal.

| mIRI Total | Frequency | Cumulative Frequency |
| --- | --- | --- |
| 29 | 1 | 0.5 |
| 33 | 1 | 0.9 |
| 35 | 1 | 1.4 |
| 36 | 4 | 3.2 |
| 37 | 3 | 4.6 |
| 38 | 7 | 7.9 |
| 39 | 1 | 8.3 |
| 40 | 1 | 8.8 |
| 41 | 7 | 12.0 |
| 42 | 8 | 15.7 |
| 43 | 10 | 20.4 |
| 44 | 8 | 24.1 |
| 45 | 6 | 26.9 |
| 46 | 2 | 27.8 |
| 47 | 7 | 31.0 |
| 48 | 6 | 33.8 |
| 49 | 9 | 38.0 |
| 50 | 6 | 40.7 |
| 51 | 6 | 43.5 |
| 52 | 5 | 45.8 |
| 53 | 10 | 50.5 |
| 54 | 11 | 55.6 |
| 55 | 8 | 59.3 |
| 56 | 6 | 62.0 |
| 57 | 6 | 64.8 |
| 58 | 8 | 68.5 |
| 59 | 7 | 71.8 |
| 60 | 7 | 75.0 |
| 61 | 7 | 78.2 |
| 62 | 7 | 81.5 |
| 63 | 2 | 82.4 |
| 64 | 10 | 87.0 |
| 65 | 4 | 88.9 |
| 66 | 7 | 92.1 |
| 67 | 3 | 93.5 |
| 68 | 2 | 94.4 |
| 69 | 3 | 95.8 |
| 70 | 9 | 100.0 |

**Table S2: Mean and standard deviation (SD) mIRI Total scores in controls by sex and age (per decade), including the number (N) of individuals in each group.**

|  | Controls | | | Females | | | Males | | |
| --- | --- | --- | --- | --- | --- | --- | --- | --- | --- |
|  | N | M | SD | N | M | SD | N | M | SD |
| 18-29.9 | 26 | 52.8 | 7.4 | 13 | 53.9 | 8.0 | 13 | 51.7 | 6.8 |
| 30-39.9 | 54 | 52.3 | 9.3 | 33 | 54.9 | 8.4 | 21 | 48.2 | 9.4 |
| 40-49.9 | 60 | 51.9 | 10.4 | 30 | 52.6 | 10.1 | 30 | 51.2 | 10.8 |
| 50-59.9 | 39 | 55.6 | 9.0 | 29 | 55.4 | 9.4 | 10 | 56.3 | 8.4 |
| 60-69.9 | 29 | 52.7 | 10.2 | 21 | 53.9 | 9.5 | 8 | 49.8 | 12.1 |
| 70+ | 8 | 54.8 | 10.2 | 3 | 53.0 | 17.3 | 5 | 55.8 | 5.4 |

Table S3: Adjusted mean differences in the mIRI Total score between groups with p-values (significant in bold) and 95% bootstrapped confidence intervals.

|  |  | Control | | *C9orf72* | | | | | | | *GRN* | | | | | | *MAPT* | | | | | |
| --- | --- | --- | --- | --- | --- | --- | --- | --- | --- | --- | --- | --- | --- | --- | --- | --- | --- | --- | --- | --- | --- | --- |
|  |  |  |  | 0 | | | 0.5 | | 1+ | | 0 | | 0.5 | | 1+ | | 0 | | 0.5 | | 1+ | |
| Control | |  |  | -1.90 | | | -2.00 | | -11.08 | | -0.20 | | -0.72 | | -8.23 | | -1.21 | | 0.15 | | -5.97 | |
|  |  |  |  | 0.058 | | | **0.046** | | **< 0.001** | | 0.845 | | 0.474 | | **< 0.001** | | 0.227 | | 0.879 | | **< 0.001** | |
|  |  |  |  | -4.58 | 0.08 | | -8.81 | 0.09 | -18.46 | -12.91 | -2.18 | 1.78 | -6.92 | 3.22 | -18.10 | -11.14 | -5.66 | 1.34 | -5.82 | 6.80 | -23.97 | -12.12 |
| *C9orf72* | 0 |  |  |  | |  | -0.94 | | -8.32 | | 1.63 | | 0.15 | | -6.42 | | 0.05 | | 0.82 | | -5.08 | |
|  |  |  |  |  | |  | 0.349 | | **< 0.001** | | 0.104 | | 0.883 | | **< 0.001** | | 0.962 | | 0.413 | | **< 0.001** | |
|  |  |  |  |  | |  | -6.80 | 2.40 | -16.60 | -10.27 | -0.42 | 4.52 | -4.88 | 5.68 | -16.15 | -8.59 | -3.73 | 3.91 | -3.82 | 9.30 | -21.89 | -9.70 |
|  | 0.5 |  |  |  | |  |  |  | -4.52 | | 1.87 | | 0.78 | | -3.69 | | 0.85 | | 1.27 | | -3.72 | |
|  |  |  |  |  | |  |  |  | **< 0.001** | | 0.062 | | 0.434 | | **< 0.001** | | 0.397 | | 0.206 | | **< 0.001** | |
|  |  |  |  |  | |  |  |  | -16.10 | -6.36 | -0.21 | 8.71 | -3.90 | 9.10 | -15.57 | -4.78 | -3.01 | 7.59 | -2.71 | 12.59 | -20.76 | -6.43 |
|  | 1+ |  |  |  | |  |  |  |  |  | 10.42 | | 4.90 | | 0.52 | | 6.36 | | 4.78 | | -0.76 | |
|  |  |  |  |  | |  |  |  |  |  | **< 0.001** | | **< 0.001** | | 0.604 | | **< 0.001** | | **< 0.001** | | 0.446 | |
|  |  |  |  |  | |  |  |  |  |  | 12.57 | 18.40 | 8.30 | 19.36 | -2.95 | 5.08 | 9.36 | 17.69 | 9.54 | 22.80 | -8.44 | 3.71 |
| *GRN* | 0 |  |  |  | |  |  |  |  | |  |  | -0.63 | | -7.87 | | -1.06 | | 0.21 | | -5.88 | |
|  |  |  |  |  | |  |  |  |  | |  |  | 0.527 | | **< 0.001** | | 0.291 | | 0.832 | | **< 0.001** | |
|  |  |  |  |  | |  |  |  |  |  |  |  | -6.78 | 3.47 | -18.02 | -10.83 | -5.60 | 1.68 | -5.67 | 7.04 | -23.80 | -11.90 |
|  | 0.5 |  |  |  | |  |  |  |  | |  | |  |  | -4.25 | | -0.10 | | 0.59 | | -4.17 | |
|  |  |  |  |  | |  |  |  |  | |  | |  |  | **< 0.001** | | 0.920 | | 0.558 | | **< 0.001** | |
|  |  |  |  |  | |  |  |  |  |  |  |  |  |  | -18.65 | -6.88 | -6.24 | 5.63 | -5.50 | 10.19 | -23.81 | -8.58 |
|  | 1+ |  |  |  | |  |  |  |  | |  | |  | |  |  | 5.21 | | 4.27 | | -1.00 | |
|  |  |  |  |  | |  |  |  |  | |  | |  | |  |  | **< 0.001** | | **< 0.001** | | 0.316 | |
|  |  |  |  |  | |  |  |  |  |  |  |  |  |  |  |  | 7.78 | 17.15 | 8.18 | 22.04 | -10.12 | 3.27 |
| *MAPT* | 0 |  |  |  | |  |  |  |  | |  | |  | |  | |  |  | 0.74 | | -4.74 | |
|  |  |  |  |  | |  |  |  |  | |  | |  | |  | |  |  | 0.461 | | **< 0.001** | |
|  |  |  |  |  | |  |  |  |  |  |  |  |  |  |  |  |  |  | -4.38 | 9.68 | -22.46 | -9.31 |
|  | 0.5 |  |  |  | |  |  |  |  | |  | |  | |  | |  | |  |  | -4.27 | |
|  |  |  |  |  | |  |  |  |  | |  | |  | |  | |  | |  |  | **< 0.001** | |
|  |  |  |  |  | |  |  |  |  |  |  |  |  |  |  |  |  |  |  |  | -27.05 | -10.02 |
|  | 1+ |  |  |  | |  |  |  |  |  |  |  |  |  |  |  |  |  |  |  |  |  |
|  |  |  |  |  | |  |  |  |  |  |  |  |  |  |  |  |  |  |  |  |  |  |
|  |  |  |  |  | |  |  |  |  |  |  |  |  |  |  |  |  |  |  |  |  |  |

**Table S4: Adjusted mean differences in the mIRI Empathic Concern (EC) subscore between groups with p-values (significant in bold) and 95% bootstrapped confidence intervals.**

|  |  | Control | | *C9orf72* | | | | | | *GRN* | | | | | | *MAPT* | | | | | |
| --- | --- | --- | --- | --- | --- | --- | --- | --- | --- | --- | --- | --- | --- | --- | --- | --- | --- | --- | --- | --- | --- |
|  |  |  |  | 0 | | 0.5 | | 1+ | | 0 | | 0.5 | | 1+ | | 0 | | 0.5 | | 1+ | |
| Control | |  |  | -1.92 | | -1.71 | | -7.04 | | -0.74 | | -0.47 | | -6.56 | | -1.09 | | 0.31 | | -4.00 | |
|  |  |  |  | 0.055 | | 0.087 | | **< 0.001** | | 0.459 | | 0.639 | | **< 0.001** | | 0.276 | | 0.759 | | **< 0.001** | |
|  |  |  |  | -2.66 | 0.03 | -4.13 | 0.28 | -7.64 | -4.31 | -1.61 | 0.73 | -3.22 | 1.98 | -8.14 | -4.40 | -2.90 | 0.83 | -3.00 | 4.12 | -10.97 | -3.76 |
| *C9orf72* | 0 |  |  |  |  | -0.51 | | -4.78 | | 1.17 | | 0.50 | | -4.70 | | 0.27 | | 0.99 | | -3.21 | |
|  |  |  |  |  |  | 0.613 | | **< 0.001** | | 0.244 | | 0.620 | | **< 0.001** | | 0.789 | | 0.323 | | **0.001** | |
|  |  |  |  |  |  | -2.98 | 1.76 | -6.57 | -2.75 | -0.60 | 2.35 | -2.05 | 3.43 | -7.02 | -2.89 | -1.77 | 2.33 | -1.84 | 5.58 | -9.74 | -2.36 |
|  | 0.5 |  |  |  | |  |  | -3.03 | | 1.28 | | 0.78 | | -3.07 | | 0.65 | | 1.18 | | -2.59 | |
|  |  |  |  |  | |  |  | **0.002** | | 0.201 | | 0.435 | | **0.002** | | 0.516 | | 0.240 | | **0.009** | |
|  |  |  |  |  |  |  |  | -6.67 | -1.43 | -0.79 | 3.77 | -1.97 | 4.58 | -7.11 | -1.57 | -1.80 | 3.58 | -1.66 | 6.63 | -9.55 | -1.33 |
|  | 1+ |  |  |  | |  | |  |  | 6.05 | | 3.59 | | -0.25 | | 4.14 | | 3.39 | | -0.72 | |
|  |  |  |  |  | |  | |  |  | **< 0.001** | | **< 0.001** | | 0.802 | | **< 0.001** | | **0.001** | | 0.471 | |
|  |  |  |  |  |  |  |  |  |  | 3.75 | 7.33 | 2.43 | 8.28 | -2.57 | 1.99 | 2.60 | 7.28 | 2.75 | 10.32 | -5.16 | 2.39 |
| *GRN* | 0 |  |  |  | |  | |  | |  |  | -0.13 | | -5.83 | | -0.58 | | 0.55 | | -3.73 | |
|  |  |  |  |  | |  | |  | |  |  | 0.893 | | **< 0.001** | | 0.560 | | 0.585 | | **< 0.001** | |
|  |  |  |  |  |  |  |  |  |  |  |  | -2.82 | 2.46 | -7.79 | -3.87 | -2.59 | 1.40 | -2.58 | 4.58 | -10.56 | -3.29 |
|  | 0.5 |  |  |  | |  | |  | |  | |  |  | -3.60 | | -0.27 | | 0.54 | | -3.04 | |
|  |  |  |  |  | |  | |  | |  | |  |  | **< 0.001** | | 0.789 | | 0.590 | | **0.002** | |
|  |  |  |  |  |  |  |  |  |  |  |  |  |  | -8.72 | -2.57 | -3.45 | 2.62 | -3.11 | 5.46 | -11.09 | -2.39 |
|  | 1+ |  |  |  | |  | |  | |  | |  | |  |  | 4.17 | | 3.47 | | -0.54 | |
|  |  |  |  |  | |  | |  | |  | |  | |  |  | **< 0.001** | | **0.001** | | 0.586 | |
|  |  |  |  |  |  |  |  |  |  |  |  |  |  |  |  | 2.77 | 7.69 | 2.97 | 10.68 | -5.04 | 2.85 |
| *MAPT* | 0 |  |  |  | |  | |  | |  | |  | |  | |  |  | 0.81 | | -3.18 | |
|  |  |  |  |  | |  | |  | |  | |  | |  | |  |  | 0.420 | | **0.001** | |
|  |  |  |  |  |  |  |  |  |  |  |  |  |  |  |  |  |  | -2.28 | 5.47 | -10.24 | -2.43 |
|  | 0.5 |  |  |  | |  | |  | |  | |  | |  | |  | |  |  | -3.07 | |
|  |  |  |  |  | |  | |  | |  | |  | |  | |  | |  |  | **0.002** | |
|  |  |  |  |  |  |  |  |  |  |  |  |  |  |  |  |  |  |  |  | -12.98 | -2.87 |
|  | 1+ |  |  |  | |  | |  | |  | |  | |  | |  | |  | |  |  |
|  |  |  |  |  | |  | |  | |  | |  | |  | |  | |  | |  |  |
|  |  |  |  |  |  |  |  |  |  |  |  |  |  |  |  |  |  |  |  |  |  |

Table S5: Adjusted mean differences in the mIRI Perspective Taking (PT) subscore between groups with p-values (significant in bold) and 95% bootstrapped confidence intervals.

|  |  | Control | | *C9orf72* | | | | | | *GRN* | | | | | | *MAPT* | | | | | |
| --- | --- | --- | --- | --- | --- | --- | --- | --- | --- | --- | --- | --- | --- | --- | --- | --- | --- | --- | --- | --- | --- |
|  |  |  |  | 0 | | 0.5 | | 1+ | | 0 | | 0.5 | | 1+ | | 0 | | 0.5 | | 1+ | |
| Control | |  |  | -1.39 | | -1.83 | | -12.91 | | 0.42 | | -0.81 | | -8.53 | | -1.08 | | -0.04 | | -7.17 | |
|  |  |  |  | 0.163 | | 0.068 | | **< 0.001** | | 0.677 | | 0.416 | | **< 0.001** | | 0.279 | | 0.970 | | **< 0.001** | |
|  |  |  |  | -2.25 | 0.38 | -5.23 | 0.18 | -11.17 | -8.23 | -0.90 | 1.39 | -4.20 | 1.74 | -10.27 | -6.43 | -3.15 | 0.91 | -3.59 | 3.45 | -13.60 | -7.76 |
| *C9orf72* | 0 |  |  |  |  | -1.10 | | -10.21 | | 1.63 | | -0.19 | | -6.92 | | -0.16 | | 0.47 | | -6.29 | |
|  |  |  |  |  |  | 0.273 | | **< 0.001** | | 0.104 | | 0.851 | | **< 0.001** | | 0.869 | | 0.641 | | **< 0.001** | |
|  |  |  |  |  |  | -4.43 | 1.25 | -10.45 | -7.09 | -0.24 | 2.60 | -3.39 | 2.79 | -9.52 | -5.32 | -2.41 | 2.04 | -2.78 | 4.51 | -12.78 | -6.71 |
|  | 0.5 |  |  |  |  |  |  | -4.85 | | 1.94 | | 0.64 | | -3.57 | | 0.84 | | 1.09 | | -4.13 | |
|  |  |  |  |  |  |  |  | **< 0.001** | | 0.052 | | 0.520 | | **< 0.001** | | 0.400 | | 0.276 | | **< 0.001** | |
|  |  |  |  |  |  |  |  | -10.09 | -4.28 | -0.02 | 5.55 | -2.64 | 5.22 | -9.03 | -2.63 | -1.86 | 4.67 | -1.96 | 6.86 | -12.03 | -4.29 |
|  | 1+ |  |  |  |  |  | |  |  | 12.48 | | 5.20 | | 1.23 | | 7.25 | | 5.16 | | -0.64 | |
|  |  |  |  |  |  |  | |  |  | **< 0.001** | | **< 0.001** | | 0.220 | | **< 0.001** | | **< 0.001** | | 0.521 | |
|  |  |  |  |  |  |  |  |  |  | 8.38 | 11.51 | 5.28 | 11.66 | -0.81 | 3.52 | 6.26 | 10.90 | 5.98 | 13.30 | -3.95 | 2.00 |
| *GRN* | 0 |  |  |  |  |  | |  | |  |  | -0.95 | | -8.41 | | -1.28 | | -0.17 | | -7.23 | |
|  |  |  |  |  |  |  | |  | |  |  | 0.341 | | **< 0.001** | | 0.202 | | 0.864 | | **< 0.001** | |
|  |  |  |  |  |  |  |  |  |  |  |  | -4.51 | 1.56 | -10.60 | -6.59 | -3.46 | 0.73 | -3.88 | 3.25 | -13.88 | -7.96 |
|  | 0.5 |  |  |  |  |  | |  | |  | |  |  | -4.16 | | 0.06 | | 0.51 | | -4.59 | |
|  |  |  |  |  |  |  | |  | |  | |  |  | **< 0.001** | | 0.951 | | 0.608 | | **< 0.001** | |
|  |  |  |  |  |  |  |  |  |  |  |  |  |  | -10.48 | -3.76 | -3.39 | 3.62 | -3.28 | 5.61 | -13.49 | -5.41 |
|  | 1+ |  |  |  |  |  | |  | |  | |  | |  |  | 5.37 | | 4.22 | | -1.34 | |
|  |  |  |  |  |  |  | |  | |  | |  | |  |  | **< 0.001** | | **< 0.001** | | 0.179 | |
|  |  |  |  |  |  |  |  |  |  |  |  |  |  |  |  | 4.59 | 9.87 | 4.44 | 12.13 | -5.72 | 1.07 |
| *MAPT* | 0 |  |  |  |  |  | |  | |  | |  | |  | |  |  | 0.52 | | -5.53 | |
|  |  |  |  |  |  |  | |  | |  | |  | |  | |  |  | 0.604 | | **< 0.001** | |
|  |  |  |  |  |  |  |  |  |  |  |  |  |  |  |  |  |  | -2.93 | 5.03 | -12.94 | -6.17 |
|  | 0.5 |  |  |  |  |  | |  | |  | |  | |  | |  | |  |  | -4.69 | |
|  |  |  |  |  |  |  | |  | |  | |  | |  | |  | |  |  | **< 0.001** | |
|  |  |  |  |  |  |  |  |  |  |  |  |  |  |  |  |  |  |  |  | -15.05 | -6.18 |
|  | 1+ |  |  |  |  |  |  |  |  |  |  |  |  |  |  |  |  |  |  |  |  |
|  |  |  |  |  |  |  |  |  |  |  |  |  |  |  |  |  |  |  |  |  |  |
|  |  |  |  |  |  |  |  |  |  |  |  |  |  |  |  |  |  |  |  |  |  |

Table S6: Adjusted mean differences in the mIRI Total score, Empathic Concern (EC) subscore, and Perspective Taking (PT) subscore between phenotypic groups with p-values (significant in bold) and 95% bootstrapped confidence intervals.

|  | **mIRI Total** | | **mIRI EC** | | **mIRI PT** | |
| --- | --- | --- | --- | --- | --- | --- |
| **Controls vs bvFTD** | -13.44 | | -9.59 | | -15.04 | |
|  | **< 0.001** | | **< 0.001** | | **< 0.001** | |
|  | -20.93 | -15.6 | -9.37 | -6.19 | -11.85 | -9.12 |
| **Controls vs PPA** | -4.11 | | -2.70 | | -4.35 | |
|  | **< 0.001** | | **0.007** | | **< 0.001** | |
|  | -13.45 | 4.77 | -5.88 | -0.93 | -8.28 | -3.13 |
| **Controls vs FTD-ALS** | -7.40 | | -3.96 | | -6.72 | |
|  | **< 0.001** | | **< 0.001** | | **< 0.001** | |
|  | -21.04 | -12.23 | -8.00 | -2.70 | -14.58 | 7.99 |
| **bvFTD vs PPA** | 3.76 | | 3.06 | | 3.45 | |
|  | **< 0.001** | | **0.002** | | **0.001** | |
|  | 4.38 | 13.93 | 1.57 | 7.18 | 2.07 | 7.49 |
| **bvFTD vs FTD-ALS** | 0.68 | | 1.67 | | -0.47 | |
|  | 0.494 | | 0.095 | | 0.641 | |
|  | -3.06 | 6.33 | -0.42 | 5.29 | -4.15 | 2.56 |
| **PPA vs FTD-ALS** | -2.51 | | -1.08 | | -2.78 | |
|  | **0.012** | | 0.280 | | **0.005** | |
|  | -13.41 | -1.64 | -5.47 | 1.58 | -9.51 | -1.64 |

Table S7: Results of VBM analysis showing anatomical regions associated with the mIRI Total scores across genetic groups.

| **Genetic group** | **Region** | **Cluster** | **T** | **Peak** | | **Co-ordinates (mm)** | | |
| --- | --- | --- | --- | --- | --- | --- | --- | --- |
|  |  |  |  | **FWE** | **Unc.** | **x** | **y** | **z** |
| *C9orf72* | Left orbitofrontal gyrus | 17 | 3.42 | 0.983 | <0.001 | -15 | 24 | -15 |
| *GRN* | Left superior, middle frontal and orbitofrontal gyri and frontal pole | 8246 | 5.73 | <0.001 | <0.001 | -18 | 58 | 10 |
|  | Left angular and supramarginal gyrus | 406 | 5.19 | 0.006 | <0.001 | -44 | -51 | 32 |
|  | Right inferior frontal gyrus, frontal operculum, insula and orbitofrontal gyrus | 849 | 4.87 | 0.025 | <0.001 | 48 | 33 | -6 |
|  | Right angular and supramarginal gyrus and superior and middle temporal gyrus | 2889 | 4.82 | 0.030 | <0.001 | 63 | -52 | 21 |
|  | Left thalamus | 1235 | 4.68 | 0.053 | <0.001 | -3 | -8 | 10 |
|  | Right caudate | 994 | 4.57 | 0.081 | <0.001 | 12 | 10 | 15 |
|  | Left angular gyrus, middle and superior temporal gyrus | 355 | 4.41 | 0.146 | <0.001 | -54 | -64 | 26 |
|  | Left fusiform gyrus | 645 | 4.33 | 0.192 | <0.001 | -36 | -90 | -18 |
|  | Left precentral gyrus and superior frontal gyrus | 711 | 4.31 | 0.206 | <0.001 | -16 | -12 | 72 |
|  | Left middle frontal gyrus and inferior frontal gyrus | 186 | 4.26 | 0.242 | <0.001 | -48 | 28 | 22 |
|  | Right superior and middle frontal gyrus | 71 | 4.19 | 0.296 | <0.001 | 20 | 39 | 38 |
|  | Right precuneus and right superior parietal lobule | 241 | 4.09 | 0.393 | <0.001 | 10 | -64 | 45 |
|  | Right frontal pole, superior frontal gyrus and orbitofrontal cortex | 269 | 4.01 | 0.487 | <0.001 | 15 | 60 | -4 |
|  | Right occipital gyrus, angular gyrus and middle temporal gyrus | 272 | 4 | 0.493 | <0.001 | 42 | -68 | 12 |
|  | Left middle and inferior temporal gyrus | 433 | 4 | 0.501 | <0.001 | -56 | -44 | -21 |
|  | Left temporal gyrus | 214 | 3.99 | 0.511 | <0.001 | -51 | -8 | -24 |
|  | Left precuneus and posterior cingulate gyrus | 287 | 3.96 | 0.542 | <0.001 | -6 | -56 | 3 |
|  | Right precentral and postcentral gyri | 217 | 3.87 | 0.654 | <0.001 | 42 | -12 | 60 |
|  | Left supplementary motor cortex and middle cingulate gyrus | 223 | 3.86 | 0.663 | <0.001 | -8 | -6 | 48 |
|  | Right middle and superior frontal gyrus | 43 | 3.86 | 0.668 | <0.001 | 22 | 51 | 14 |
|  | Right orbitofrontal cortex and anterior insula | 138 | 3.85 | 0.678 | <0.001 | 20 | 20 | -20 |
|  | Left lingual gyrus and precuneus | 63 | 3.84 | 0.682 | <0.001 | -18 | -60 | 0 |
|  | Left caudate | 485 | 3.84 | 0.686 | <0.001 | -10 | 16 | 9 |
|  | Left precentral and postcentral gyri and precuneus | 573 | 3.83 | 0.703 | <0.001 | 8 | -38 | 72 |
|  | Right middle frontal gyrus | 58 | 3.8 | 0.728 | <0.001 | 44 | 30 | 42 |
|  | Right superior frontal gyrus | 14 | 3.79 | 0.738 | <0.001 | 16 | 30 | 58 |
|  | Right cerebellum | 48 | 3.77 | 0.760 | <0.001 | 20 | -38 | -44 |
|  | Left superior parietal lobule, postcentral, supramarginal and angular gyrus | 195 | 3.76 | 0.778 | <0.001 | -36 | -42 | 51 |
|  | Right supplementary motor cortex and superior frontal gyrus | 28 | 3.74 | 0.799 | <0.001 | 10 | 22 | 52 |
|  | Right superior frontal gyrus | 17 | 3.71 | 0.825 | <0.001 | 20 | 39 | 51 |
|  | Left anterior cingulate gyrus | 69 | 3.71 | 0.825 | <0.001 | -3 | -42 | 21 |
|  | Left hippocampus, parahippocampal gyrus and thalamus | 131 | 3.71 | 0.831 | <0.001 | -20 | -36 | -8 |
|  | Right middle frontal gyrus | 16 | 3.62 | 0.903 | <0.001 | 38 | 14 | 42 |
|  | Left lingual gyrus, posterior cingulate and precuneus | 102 | 3.59 | 0.921 | <0.001 | 9 | -51 | 2 |
|  | Left inferior and middle temporal gyrus and fusiform gyrus | 202 | 3.58 | 0.924 | <0.001 | 51 | -56 | -12 |
|  | Left superior frontal gyrus | 34 | 3.57 | 0.932 | <0.001 | -10 | 45 | 36 |
|  | Right fusiform and parahippocampal gyrus | 30 | 3.57 | 0.933 | <0.001 | 36 | -38 | -18 |
|  | Right precentral and superior frontal gyrus | 30 | 3.56 | 0.937 | <0.001 | 52 | 4 | 46 |
|  | Left postcentral gyrus and superior parietal lobule | 20 | 3.56 | 0.938 | <0.001 | -32 | -39 | 64 |
|  | Left postcentral gyrus, precuneus and superior parietal lobule | 10 | 3.56 | 0.938 | <0.001 | -8 | -45 | 74 |
|  | Left pre and post central gyri and precuneus | 17 | 3.54 | 0.946 | <0.001 | -9 | -36 | 62 |
|  | Right superior frontal gyrus and anterior cingulate gyrus | 117 | 3.53 | 0.950 | <0.001 | 12 | 38 | 22 |
|  | Left superior parietal lobule, supramarginal and angular gyrus | 35 | 3.52 | 0.954 | <0.001 | -40 | -45 | 36 |
|  | Right entorhinal area, temporal pole, orbitofrontal cortex and amygdala | 15 | 3.52 | 0.957 | <0.001 | 15 | 4 | -32 |
|  | Left supplementary motor cortex and superior frontal gyrus | 19 | 3.51 | 0.958 | <0.001 | -9 | 20 | 51 |
|  | Left middle frontal gyrus | 10 | 3.49 | 0.966 | <0.001 | -39 | 50 | 16 |
|  | Left medial frontal cortex, frontal pole, superior and middle frontal gyrus | 11 | 3.47 | 0.972 | <0.001 | -3 | 62 | -4 |
|  | Right middle frontal gyrus, precentral gyrus and operculum | 15 | 3.47 | 0.972 | <0.001 | 44 | 12 | 33 |
|  | Left superior and middle frontal gyrus | 17 | 3.47 | 0.972 | <0.001 | -27 | 14 | 56 |
|  | Right fusiform gyrus, occipital gyrus and cerebellum | 27 | 3.47 | 0.973 | <0.001 | 39 | -72 | -15 |
|  | Right precuneus, cuneus, lingual gyrus, calcarine cortex and posterior cingulate | 21 | 3.46 | 0.974 | <0.001 | 15 | -56 | 10 |
|  | Left supplementary motor cortex and superior frontal gyrus | 25 | 3.43 | 0.981 | <0.001 | -3 | 2 | 62 |
|  | Left pre and post central gyri | 14 | 3.42 | 0.983 | <0.001 | -10 | -30 | 76 |
|  | Left temporal gyrus | 14 | 3.42 | 0.984 | <0.001 | -60 | -27 | -12 |
|  | Right occipital gyrus, angular gyrus and superior parietal lobule | 12 | 3.42 | 0.984 | <0.001 | 38 | -68 | 32 |
|  | Left hippocampus | 13 | 3.41 | 0.986 | <0.001 | -34 | -28 | -6 |
|  | Left supplementary motor cortex and superior frontal gyrus | 32 | 3.41 | 0.986 | <0.001 | -4 | 16 | 62 |
|  | Right thalamus and hippocampus | 109 | 3.4 | 0.987 | <0.001 | 22 | -33 | -3 |
|  | Right inferior and middle temporal gyrus, and inferior occipital gyrus | 14 | 3.4 | 0.987 | <0.001 | 52 | -58 | 0 |
|  | Right supramarginal gyrus, postcentral gyrus and parietal operculum | 55 | 3.39 | 0.989 | <0.001 | 57 | -24 | 38 |
|  | Right inferior occipital gyrus | 32 | 3.36 | 0.992 | <0.001 | 46 | -81 | -6 |
|  | Left calcarine cortex and cuneus | 20 | 3.36 | 0.993 | <0.001 | -8 | -78 | 15 |
|  | Right orbitofrontal cortex and frontal pole | 14 | 3.33 | 0.995 | <0.001 | 6 | 60 | -21 |
|  | Left posterior cingulate | 14 | 3.33 | 0.995 | <0.001 | -2 | -33 | 44 |
|  | Right supramarginal gyrus | 14 | 3.32 | 0.996 | 0.001 | 54 | -28 | 48 |
|  | Left pre and post central gyri | 15 | 3.31 | 0.996 | 0.001 | -52 | -9 | 45 |
|  | Right precuneus | 20 | 3.25 | 0.999 | 0.001 | 10 | -57 | 20 |
|  | Right middle and superior frontal gyrus and orbitofrontal gyrus | 13 | 3.24 | 0.999 | 0.001 | 33 | 51 | -3 |
|  | Right precentral and postcentral gyri and superior parietal lobule | 10 | 3.22 | 0.999 | 0.001 | 33 | -34 | 64 |
| *MAPT* | Left amygdala, hippocampus, temporal pole, orbitofrontal cortex, insula, parahippocampal and fusiform gyri | 5982 | 6.07 | <0.001 | <0.001 | -27 | -4 | -27 |
|  | Right amygdala, hippocampus, temporal pole, orbitofrontal cortex, insula, parahippocampal and fusiform gyri | 4824 | 4.75 | 0.041 | <0.001 | 28 | -4 | -27 |
|  | Left temporal gyrus | 52 | 4.21 | 0.281 | <0.001 | -54 | -21 | -16 |
|  | Left fusiform gyrus and lingual gyrus | 43 | 4.17 | 0.313 | <0.001 | -12 | -90 | -24 |
|  | Left middle and inferior temporal gyrus, and fusiform gyrus | 26 | 4.08 | 0.404 | <0.001 | 50 | -32 | -18 |
|  | Left putamen, caudate and nucleus accumbens | 205 | 3.79 | 0.740 | <0.001 | -10 | 8 | -8 |
|  | Right inferior temporal and occipital gyrus, and fusiform gyrus | 83 | 3.61 | 0.909 | <0.001 | 45 | -57 | -12 |
|  | Right temporal pole and middle temporal gyrus | 47 | 3.6 | 0.911 | <0.001 | 50 | 14 | -30 |
|  | Right putamen and caudate | 119 | 3.52 | 0.957 | <0.001 | 10 | 8 | -8 |
|  | Left orbitofrontal cortex, putamen, nucleus accumbens, caudate | 14 | 3.47 | 0.973 | <0.001 | -16 | 21 | -15 |
|  | Left thalamus and caudate | 18 | 3.3 | 0.997 | 0.001 | -6 | -2 | -2 |
|  | Right middle temporal gyrus | 32 | 3.29 | 0.997 | 0.001 | 62 | -9 | -20 |
|  | Right subcallosal area and nucleus accumbens | 10 | 3.27 | 0.998 | 0.001 | 4 | 14 | -15 |

Table S8: VBM analysis showing the neuroanatomical regions associated with the mIRI Perspective Taking (PT) subscore across genetic groups.

| **Genetic group** | **Region** | **Cluster** | **T** | **Peak** | | **Co-ordinates (mm)** | | |
| --- | --- | --- | --- | --- | --- | --- | --- | --- |
|  |  |  |  | **FWE** | **Unc.** | **x** | **y** | **z** |
| *C9orf72* | Left orbitofrontal cortex | 12 | 3.52 | 0.953 | <0.001 | -39 | 30 | -21 |
| *GRN* | Left superior and middle frontal gyri, orbitofrontal cortex and frontal pole | 8801 | 5.96 | <0.001 | <0.001 | -18 | 58 | 10 |
|  | Left angular and supramarginal gyri, and superior parietal lobule | 258 | 5.35 | 0.003 | <0.001 | -42 | -54 | 33 |
|  | Right angular and supramarginal gyri, postcentral gyrus, parietal operculum and superior parietal lobule | 1090 | 4.71 | 0.047 | <0.001 | 42 | -38 | 45 |
|  | Left angular and supramarginal gyri and superior and middle temporal gyri, and middle occipital gyrus | 502 | 4.69 | 0.051 | <0.001 | -40 | -56 | 14 |
|  | Right angular and supramarginal gyrus, and middle and superior temporal gyrus | 703 | 4.54 | 0.091 | <0.001 | 63 | -52 | 21 |
|  | Right middle and superior frontal gyri, | 113 | 4.5 | 0.105 | <0.001 | 18 | 38 | 38 |
|  | Left thalamus | 579 | 4.43 | 0.136 | <0.001 | -3 | -6 | 12 |
|  | Right caudate, putamen, and thalamus | 863 | 4.37 | 0.166 | <0.001 | 10 | 10 | 14 |
|  | Right precuneus, superior parietal lobule, angular gyrus, and occipital gyrus | 317 | 4.37 | 0.168 | <0.001 | 10 | -64 | 46 |
|  | Left inferior and middle temporal gyrus | 478 | 4.33 | 0.190 | <0.001 | -56 | -44 | -22 |
|  | Left lingual gyrus, posterior cingulate and insula, calcarine cortex, and precuneus and hippocampus | 1441 | 4.29 | 0.216 | <0.001 | -8 | -54 | 3 |
|  | Right inferior frontal gyrus, orbitofrontal cortex, frontal operculum, and anterior insula | 549 | 4.25 | 0.246 | <0.001 | 46 | 34 | -9 |
|  | Left angular and supramarginal gyrus, and superior parietal lobule | 137 | 4.23 | 0.262 | <0.001 | -56 | -54 | 38 |
|  | Left temporal pole | 234 | 4.2 | 0.285 | <0.001 | -51 | -8 | -24 |
|  | Right hippocampus, parahippocampal gyrus, fusiform gyrus, and thalamus | 367 | 4.19 | 0.301 | <0.001 | 36 | -28 | -12 |
|  | Right superior and middle frontal gyrus | 60 | 4.15 | 0.331 | <0.001 | 22 | 52 | 14 |
|  | Right superior and middle frontal gyrus | 153 | 4.11 | 0.378 | <0.001 | 45 | 28 | 40 |
|  | Right frontal pole, superior frontal gyrus, and orbitofrontal cortex | 517 | 4.09 | 0.398 | <0.001 | 15 | 60 | -4 |
|  | Right superior frontal gyrus | 109 | 4.07 | 0.417 | <0.001 | 12 | 51 | 40 |
|  | Right superior frontal gyrus and supplementary motor cortex | 156 | 4.03 | 0.455 | <0.001 | 10 | 24 | 52 |
|  | Right temporal pole | 395 | 4.03 | 0.456 | <0.001 | 51 | -12 | -21 |
|  | Right superior parietal lobule, postcentral gyrus, angular and supramarginal gyrus | 173 | 4.01 | 0.483 | <0.001 | -28 | -44 | 50 |
|  | Left middle and superior frontal gyrus | 184 | 3.9 | 0.608 | <0.001 | -27 | 14 | 54 |
|  | Left supplementary motor cortex and superior frontal gyrus | 149 | 3.9 | 0.618 | <0.001 | -9 | 20 | 51 |
|  | Left precentral and postcentral gyri | 61 | 3.85 | 0.675 | <0.001 | -52 | -10 | 46 |
|  | Left orbitofrontal cortex | 33 | 3.82 | 0.712 | <0.001 | -30 | 48 | 2 |
|  | Left middle and inferior frontal gyrus | 62 | 3.81 | 0.717 | <0.001 | -48 | 28 | 22 |
|  | Left anterior cingulate and precentral gyrus | 248 | 3.8 | 0.729 | <0.001 | -2 | -32 | 44 |
|  | Left caudate, putamen, and thalamus | 668 | 3.78 | 0.757 | <0.001 | -6 | 14 | 3 |
|  | Left temporal pole | 25 | 3.78 | 0.758 | <0.001 | -58 | -27 | -14 |
|  | Left fusiform gyrus, and cerebellum | 77 | 3.77 | 0.770 | <0.001 | -36 | -90 | -18 |
|  | Left anterior cingulate | 148 | 3.76 | 0.772 | <0.001 | -3 | -42 | 21 |
|  | Left superior frontal gyrus | 157 | 3.76 | 0.772 | <0.001 | -10 | 42 | 39 |
|  | Left supplementary motor cortex and cingulate | 204 | 3.75 | 0.789 | <0.001 | -6 | -6 | 48 |
|  | Left angular gyrus and middle temporal gyrus | 87 | 3.65 | 0.881 | <0.001 | 42 | -70 | 18 |
|  | Right superior frontal gyrus, supplementary motor cortex and anterior cingulate | 169 | 3.64 | 0.884 | <0.001 | 12 | 38 | 22 |
|  | Right orbitofrontal cortex | 57 | 3.64 | 0.887 | <0.001 | 10 | 50 | -18 |
|  | Right middle and superior frontal gyrus, and orbitofrontal cortex | 188 | 3.63 | 0.893 | <0.001 | 33 | 51 | -3 |
|  | Left planum polare and central operculum | 26 | 3.61 | 0.907 | <0.001 | -60 | -24 | 12 |
|  | Left superior frontal gyrus and precentral gyrus | 61 | 3.61 | 0.909 | <0.001 | -16 | -12 | 70 |
|  | Left inferior occipital gyrus, and fusiform gyrus | 31 | 3.6 | 0.915 | <0.001 | -39 | -74 | -10 |
|  | Left middle and superior frontal gyrus, and precentral gyrus | 212 | 3.53 | 0.950 | <0.001 | -28 | -4 | 51 |
|  | Left middle and inferior temporal gyrus, and inferior occipital gyrus | 112 | 3.52 | 0.953 | <0.001 | 50 | -72 | -4 |
|  | Left superior frontal gyrus and supplementary motor cortex | 53 | 3.52 | 0.954 | <0.001 | -6 | 32 | 42 |
|  | Right orbitofrontal cortex | 27 | 3.52 | 0.954 | <0.001 | 6 | 58 | -20 |
|  | Right cerebellum | 20 | 3.52 | 0.955 | <0.001 | 20 | -36 | -44 |
|  | Left superior frontal gyrus and supplementary motor cortex | 20 | 3.51 | 0.957 | <0.001 | -10 | 21 | 63 |
|  | Left postcentral gyrus and superior parietal lobule | 17 | 3.48 | 0.969 | <0.001 | -32 | -39 | 64 |
|  | Right precuneus, cuneus, calcarine cortex and lingual gyrus | 98 | 3.47 | 0.970 | <0.001 | 15 | -56 | 10 |
|  | Right superior frontal gyrus | 12 | 3.44 | 0.979 | <0.001 | 18 | 39 | 50 |
|  | Left central operculum and insula | 64 | 3.43 | 0.981 | <0.001 | -38 | -14 | 18 |
|  | Left superior and middle frontal gyrus | 11 | 3.42 | 0.983 | <0.001 | -16 | 32 | 40 |
|  | Left orbitofrontal cortex | 21 | 3.41 | 0.985 | <0.001 | 0 | 50 | -24 |
|  | Left precentral and postcentral gyri | 18 | 3.41 | 0.986 | <0.001 | -45 | -16 | 42 |
|  | Left precuneus and posterior cingulate | 12 | 3.4 | 0.987 | <0.001 | -12 | -52 | 40 |
|  | Right precuneus | 72 | 3.39 | 0.988 | <0.001 | 10 | -58 | 22 |
|  | Right lingual gyrus and posterior cingulate | 24 | 3.38 | 0.990 | <0.001 | 14 | -46 | -2 |
|  | Right middle frontal gyrus | 10 | 3.38 | 0.990 | <0.001 | -42 | 22 | 45 |
|  | Left supplementary motor cortex | 14 | 3.38 | 0.990 | <0.001 | -3 | 2 | 62 |
|  | Right superior frontal gyrus and cingulate | 23 | 3.38 | 0.990 | <0.001 | 8 | 46 | -2 |
|  | Right precentral and postcentral gyri | 33 | 3.36 | 0.993 | <0.001 | 44 | -10 | 57 |
|  | Right middle and inferior temporal gyrus | 16 | 3.34 | 0.994 | <0.001 | 68 | -48 | -3 |
|  | Left cuneus and calcarine cortex | 11 | 3.34 | 0.994 | <0.001 | -6 | -76 | 16 |
|  | Right postcentral gyrus | 18 | 3.34 | 0.994 | <0.001 | 8 | -39 | 70 |
|  | Right anterior insula | 32 | 3.33 | 0.995 | <0.001 | 32 | 20 | -10 |
|  | Right orbitofrontal cortex and insula | 11 | 3.31 | 0.996 | 0.001 | 28 | 28 | -10 |
|  | Left precentral and postcentral gyri | 26 | 3.31 | 0.996 | 0.001 | -50 | -3 | 28 |
|  | Right angular gyrus | 25 | 3.26 | 0.998 | 0.001 | 54 | -60 | 21 |
|  | Left caudate | 13 | 3.25 | 0.999 | 0.001 | -18 | -9 | 21 |
|  | Left inferior temporal gyrus | 32 | 3.24 | 0.999 | 0.001 | 58 | -54 | -12 |
| *MAPT* | Left amygdala, hippocampus, fusiform and middle temporal gyri, temporal pole, and orbitofrontal cortex | 9908 | 7.31 | <0.001 | <0.001 | -27 | -6 | -27 |
|  | Right amygdala, hippocampus, putamen, fusiform gyri, temporal pole and orbitofrontal cortex | 7394 | 5.35 | 0.003 | <0.001 | 26 | -8 | -15 |
|  | Left temporal gyri | 78 | 4.72 | 0.045 | <0.001 | -54 | -21 | -16 |
|  | Left inferior and middle temporal, and fusiform gyrus. | 26 | 4.07 | 0.419 | <0.001 | 50 | -32 | -18 |
|  | Left caudate, putamen, and anterior cingulate | 571 | 3.8 | 0.736 | <0.001 | -3 | 9 | -2 |
|  | Right middle and superior temporal gyrus | 77 | 3.53 | 0.952 | <0.001 | 62 | -9 | -20 |
|  | Left inferior occipital and temporal gyrus, and fusiform gyrus | 47 | 3.43 | 0.981 | <0.001 | 44 | -57 | -10 |
|  | Right parahippocampal gyrus, hippocampus and fusiform gyrus | 17 | 3.28 | 0.997 | 0.001 | 28 | -34 | -15 |

Table S9: VBM analysis displaying the neuroanatomical regions associated with the mIRI Empathic Concern (EC) subscore across genetic groups.

| **Genetic group** | **Region** | **Cluster** | **T** | **Peak** | | **Co-ordinates (mm)** | | |
| --- | --- | --- | --- | --- | --- | --- | --- | --- |
|  |  |  |  | **FWE** | **Unc.** | **x** | **y** | **z** |
| *C9orf72* | Right putamen and insula | 285 | 4.48 | 0.114 | <0.001 | 32 | -15 | -6 |
|  | Left putamen and insula | 203 | 3.88 | 0.639 | <0.001 | -26 | -4 | 2 |
|  | Right fusiform, parahippocampal, entorhinal and inferior temporal gyrus | 11 | 3.7 | 0.834 | <0.001 | 22 | -9 | -42 |
|  | Right inferior and middle temporal gyrus | 23 | 3.53 | 0.949 | <0.001 | 56 | -46 | -16 |
|  | Left orbitofrontal gyrus, medial frontal cortex and anterior cingulate | 17 | 3.45 | 0.978 | <0.001 | -15 | 24 | -15 |
| *GRN* | Right inferior frontal gyrus, operculum and middle frontal gyrus | 556 | 5.01 | 0.014 | <0.001 | 56 | 34 | -4 |
|  | Left precentral and postcentral gyri, superior frontal gyrus and supplementary motor cortex | 1646 | 4.7 | 0.048 | <0.001 | -12 | -15 | 74 |
|  | Left thalamus | 958 | 4.36 | 0.173 | <0.001 | 6 | -8 | 6 |
|  | Left angular and supramarginal gyrus | 98 | 4.3 | 0.210 | <0.001 | -44 | -51 | 32 |
|  | Left middle and superior frontal gyrus | 175 | 4.29 | 0.217 | <0.001 | -27 | 45 | 26 |
|  | Left inferior frontal gyrus, orbitofrontal cortex and frontal operculum | 450 | 4.27 | 0.235 | <0.001 | -46 | 30 | -3 |
|  | Right angular, supramarginal, middle and superior temporal gyri and parietal operculum | 852 | 4.23 | 0.262 | <0.001 | 54 | -42 | 27 |
|  | Right superior parietal lobule, angular and supramarginal gyrus and postcentral gyrus | 140 | 4.12 | 0.366 | <0.001 | 42 | -40 | 50 |
|  | Left orbitofrontal cortex, caudate, cingulate and subcallosal area | 193 | 4.07 | 0.420 | <0.001 | -16 | 22 | -16 |
|  | Left fusiform gyrus, inferior temporal gyrus and cerebellum | 540 | 4.05 | 0.437 | <0.001 | -28 | -90 | -20 |
|  | Left middle and superior frontal gyrus, orbitofrontal cortex and frontal pole | 490 | 3.98 | 0.513 | <0.001 | -18 | 58 | 10 |
|  | Right anterior cingulate | 209 | 3.96 | 0.540 | <0.001 | -2 | 34 | 12 |
|  | Right orbitofrontal cortex and anterior insula | 92 | 3.96 | 0.544 | <0.001 | 20 | 20 | -20 |
|  | Right orbitofrontal cortex and medial frontal cortex | 223 | 3.96 | 0.547 | <0.001 | 2 | 48 | -26 |
|  | Right entorhinal cortex and temporal pole | 50 | 3.78 | 0.759 | <0.001 | 16 | 6 | -34 |
|  | Right inferior and middle temporal gyrus, and fusiform gyrus | 210 | 3.71 | 0.826 | <0.001 | 51 | -42 | -16 |
|  | Right middle occipital gyrus | 69 | 3.7 | 0.832 | <0.001 | 45 | -78 | 22 |
|  | Right occipital gyrus, middle temporal gyrus and angular gyrus | 40 | 3.67 | 0.862 | <0.001 | 42 | -68 | 12 |
|  | Right caudate | 207 | 3.67 | 0.863 | <0.001 | 12 | 12 | 15 |
|  | Right cerebellum, fusiform gyrus and inferior occipital gyrus | 216 | 3.65 | 0.877 | <0.001 | 38 | -81 | -24 |
|  | Left middle and inferior frontal gyrus | 76 | 3.65 | 0.877 | <0.001 | -48 | 28 | 22 |
|  | Left angular and middle occipital gyrus | 24 | 3.6 | 0.910 | <0.001 | -52 | -70 | 21 |
|  | Left angular gyrus | 39 | 3.6 | 0.913 | <0.001 | -52 | -66 | 33 |
|  | Right precentral and middle frontal gyrus | 32 | 3.57 | 0.929 | <0.001 | 52 | 6 | 46 |
|  | Right precentral and postcentral gyri | 73 | 3.51 | 0.958 | <0.001 | 42 | -12 | 60 |
|  | Right lingual gyrus and cerebellum | 67 | 3.5 | 0.962 | <0.001 | 12 | -54 | -3 |
|  | Left inferior frontal gyrus and frontal operculum | 37 | 3.5 | 0.963 | <0.001 | -56 | 20 | 10 |
|  | Left supramarginal and post central gyrus | 38 | 3.48 | 0.969 | <0.001 | -58 | -27 | 39 |
|  | Right medial frontal cortex | 18 | 3.46 | 0.974 | <0.001 | 2 | 56 | -12 |
|  | Left supramarginal and angular gyrus | 16 | 3.45 | 0.977 | <0.001 | -52 | -48 | 50 |
|  | Left superior frontal gyrus | 18 | 3.42 | 0.983 | <0.001 | -16 | 56 | 28 |
|  | Left middle and inferior temporal gyrus | 31 | 3.42 | 0.983 | <0.001 | -57 | -46 | -12 |
|  | Left lingual gyrus, calcarine cortex and precuneus | 18 | 3.41 | 0.985 | <0.001 | -16 | -62 | 0 |
|  | Right angular gyrus and superior parietal lobule | 22 | 3.37 | 0.991 | <0.001 | 38 | -58 | 44 |
|  | Right anterior cingulate | 20 | 3.35 | 0.993 | <0.001 | 3 | 22 | 32 |
|  | Left supplementary motor cortex | 28 | 3.34 | 0.994 | <0.001 | -10 | -6 | 45 |
|  | Right cerebellum | 14 | 3.33 | 0.995 | <0.001 | 18 | -39 | -44 |
|  | Right precentral and postcentral gyri | 33 | 3.32 | 0.996 | <0.001 | 32 | -21 | 70 |
|  | Right middle frontal and precentral gyrus | 12 | 3.31 | 0.996 | 0.001 | 44 | 12 | 33 |
|  | Left central operculum and planum polare | 28 | 3.3 | 0.997 | 0.001 | -54 | 0 | 2 |
|  | Right orbitofrontal cortex and medial frontal cortex | 10 | 3.21 | 0.999 | 0.001 | 6 | 24 | -24 |
|  | Left precentral and postcentral gyri | 12 | 3.21 | 0.999 | 0.001 | -40 | -16 | 60 |
| *MAPT* | Right putamen, insula, hippocampus, orbitofrontal cortex | 1021 | 4.67 | 0.056 | <0.001 | 34 | -15 | -6 |
|  | Right lingual gyrus, fusiform gyrus and cerebellum | 78 | 4.24 | 0.256 | <0.001 | -12 | -90 | -24 |
|  | Left temporal pole | 309 | 4.15 | 0.335 | <0.001 | -48 | 14 | -34 |
|  | Right putamen, insula and hippocampus | 147 | 3.91 | 0.603 | <0.001 | -32 | -16 | -4 |
|  | Left amygdala, hippocampus, parahippocampal gyrus, fusiform gyrus and temporal pole | 86 | 3.89 | 0.629 | <0.001 | -27 | -4 | -28 |
|  | Left middle and inferior temporal gyrus, fusiform gyrus and temporal pole | 94 | 3.73 | 0.810 | <0.001 | -42 | 3 | -48 |
|  | Right temporal gyri, and temporal pole | 336 | 3.68 | 0.854 | <0.001 | 45 | 2 | -44 |
|  | Left orbitofrontal cortex and gyrus rectus | 35 | 3.56 | 0.936 | <0.001 | -16 | 21 | -15 |
|  | Right amygdala, hippocampus and entorhinal area | 12 | 3.4 | 0.987 | <0.001 | 28 | -4 | -27 |
|  | Left putamen, caudate and accumbens | 26 | 3.28 | 0.998 | 0.001 | -12 | 9 | -8 |

**Figure S1: Modified Interpersonal Reactivity Index (mIRI) Empathic Concern (EC) and Perspective Taking (PT) scores in each genetic group stratified by CDR plus NACC FTLD (0 = asymptomatic, 0.5 = mildly symptomatic/prodromal, 1+ = fully symptomatic). Means and standard errors are shown. Significant differences from controls and within groups are starred (p<0.05).**

**Figure S2: Neural correlates of a) the mIRI Empathic Concern (EC) and b) the mIRI Perspective Taking (PT) subscores. Results for all three genetic groups are displayed at p<0.001 uncorrected. A study-specific T1-weighted MRI template in MNI space was used to show results. Green represents the MAPT group, yellow for the GRN group, and blue for the C9orf72 group.**

**a)**

**b)**
